# Expanding primary cells from mucoepidermoid and other salivary gland neoplasms for genetic and chemosensitivity testing

**DOI:** 10.1242/dmm.031716

**Published:** 2018-01-01

**Authors:** Ahmad M. Alamri, Xuefeng Liu, Jan K. Blancato, Bassem R. Haddad, Weisheng Wang, Xiaogang Zhong, Sujata Choudhary, Ewa Krawczyk, Bhaskar V. Kallakury, Bruce J. Davidson, Priscilla A. Furth

**Affiliations:** 1 Oncology, Georgetown University, Washington, DC 20057, USA; 2 Department of Clinical Laboratory Sciences, College of Applied Medical Sciences, King Khalid University, Abha, 61413, Saudi Arabia; 3 Pathology, Center for Cell Reprogramming, Georgetown University, Washington, DC 20057, USA; 4 Oncology, Lombardi Comprehensive Cancer Center, Georgetown University, Washington, DC 20057, USA; 5 Oncology, Georgetown University, Washington, DC 20057, USA; 6 Biostatistics, Bioinformatics and Biomathematics, Georgetown University, Washington, DC 20057, USA; 7 Pathology, Georgetown University, Washington, DC 20057, USA; 8 Pathology, Lombardi Comprehensive Cancer Center, Georgetown University, Washington, DC 20057, USA; 9 Otolaryngology - Head and Neck Surgery, MedStar Georgetown University Hospital, Washington, DC 20007, USA; 10 Oncology and Medicine, Lombardi Comprehensive Cancer Center, Georgetown University, Washington, DC 20057, USA

**Keywords:** Primary cell culture, Salivary gland neoplasms, Mucoepidermoid carcinoma, Next-generation sequencing, AKT, Drug sensitivity

## Abstract

Restricted availability of cell and animal models is a rate-limiting step for investigation of salivary gland neoplasm pathophysiology and therapeutic response. Conditionally reprogrammed cell (CRC) technology enables establishment of primary epithelial cell cultures from patient material. This study tested a translational workflow for acquisition, expansion and testing of CRC-derived primary cultures of salivary gland neoplasms from patients presenting to an academic surgical practice. Results showed that cultured cells were sufficient for epithelial cell-specific transcriptome characterization to detect candidate therapeutic pathways and fusion genes, and for screening for cancer risk-associated single nucleotide polymorphisms (SNPs) and driver gene mutations through exome sequencing. Focused study of primary cultures of a low-grade mucoepidermoid carcinoma demonstrated amphiregulin-mechanistic target of rapamycin-protein kinase B (AKT; AKT1) pathway activation, identified through bioinformatics and subsequently confirmed as present in primary tissue and preserved through different secondary 2D and 3D culture media and xenografts. Candidate therapeutic testing showed that the allosteric AKT inhibitor MK2206 reproducibly inhibited cell survival across different culture formats. By contrast, the cells appeared resistant to the adenosine triphosphate competitive AKT inhibitor GSK690693. Procedures employed here illustrate an approach for reproducibly obtaining material for pathophysiological studies of salivary gland neoplasms, and other less common epithelial cancer types, that can be executed without compromising pathological examination of patient specimens. The approach permits combined genetic and cell-based physiological and therapeutic investigations in addition to more traditional pathologic studies, and can be used to build sustainable bio-banks for future inquiries.

This article has an associated First Person interview with the first author of the paper.

## Abstract (precis)

**Summary:** Genetics and therapeutic responses of uncommon neoplasms, including salivary gland cancers, are investigatable through isolation of primary cells from patient material using conditional reprogramming cell technology without compromising pathological diagnosis.

## INTRODUCTION

Primary salivary gland neoplasms arise from both major (parotid, submandibular or sublingual gland) and minor salivary glands (Thompson, 2006). They are composed of benign and malignant tumors of different histopathological types, and exhibit a range of responses to chemotherapy (Carlson et al., 2013). Relatively uncommon, they comprise ∼5% of head and neck tumors (Speight and Barrett, 2002), with an incidence of 1.7 cases per 100,000 individuals in the United States (https://www.cancer.org/research/cancer-facts-statistics/all-cancer-facts-figures/cancer-facts-figures-2017.html). Limited case numbers and access to patient samples, coupled with scarce availability of authenticated cell lines and animal models, translate into our current relatively restricted understanding of salivary gland cancer pathophysiology and chemotherapeutic response (Warner et al., 2013). Standard treatment is surgical resection followed by postoperative radiation (Adelstein et al., 2012). Chemotherapy is reserved for recurrence, metastases, and when surgical resection is not possible (Schoenfeld et al., 2012). In addition to being a site for primary cancers, the parotid gland also hosts nonsalivary metastatic disease, particularly cutaneous squamous cell cancers, pathophysiology attributed to the presence of glandular lymph nodes (Chernock and Lewis, 2015). Sialoadenitis is a nonmalignant inflammatory condition that can present as a salivary gland neoplasm (Gupta et al., 2015).

Mucoepidermoid carcinoma (MEC) is the most common malignant salivary gland tumor (30-40% of all salivary gland malignancies) (McHugh et al., 2012). CREB-regulated transcription coactivator 1 (*CRTC1*), mastermind like transcriptional coactivator (*MAML2*), *CRTC3-MAML2* and EWS RNA binding protein 1 (*EWS*)*-*POU class 5 homeobox 1 (*POU5F1*) fusion genes are reported to be more frequent in low- as compared to high-grade MEC (Fehr et al., 2008; Möller et al., 2008; Bell and El-Naggar, 2013). Presence of *CRTC1-MAML2* is associated with overexpression of the EGF family member amphiregulin (AREG) (Wu et al., 2002; Lin et al., 2002; Stenman et al., 2014; Chen et al., 2014, 2015), and the presence of both has been correlated with longer disease-free survival (Shinomiya et al., 2016).

AREG is an epidermal growth factor (EGF) family member acting through the EGF receptor (EGFR) to activate phosphatidylinositol-4,5-bisphosphate 3-kinase (PI3K; PIK3CA) and protein kinase B (AKT; AKT1) pathways that promote cell survival and proliferation (McBryan et al., 2008; Hers et al., 2011; Edgar et al., 2014). EGFR is an ErbB family member proposed as a therapeutic biomarker in head and neck cancer (Kang et al., 2015). Activated AKT pathways are reported in many different types of salivary gland cancer (Marques et al., 2008; de Lima et al., 2009; Ettl et al., 2012; Suzuki et al., 2012), and AKT inhibitors with dissimilar mechanisms of action are available (Nitulescu et al., 2016; Xu et al., 2016). MK2206 is an oral allosteric highly selective AKT inhibitor that reduces levels of phosphorylated AKT (p-AKT), while GSK690693 is an ATP-competitive AKT inhibitor to which exposure results in increased levels of p-AKT, but reduced levels of phosphorylated glycogen synthase kinase 3 alpha/beta (p-GSK3α/β) and phosphorylated mechanistic target of rapamycin (p-mTOR) (Rhodes et al., 2008; Altomare et al., 2010). MK2206 has documented activity in phase II solid tumor clinical trials (Hirai et al., 2010; Vivanco et al., 2014; Agarwal et al., 2014; Bahrami et al., 2017); GSK690693 is a validated reference molecule for its class (Rhodes et al., 2008).

Conditionally reprogrammed cell (CRC) technology can generate primary epithelial cell cultures from normal, benign and malignant tissue from different species (Chapman et al., 2010; Liu et al., 2012, 2017; Yuan et al., 2012; Chapman et al., 2014; Ligaba et al., 2015; Timofeeva et al., 2016; Alamri et al., 2016). Here, we explored its utility for establishing primary cell cultures from different types of human salivary gland neoplasms, examined if sufficient material for next generation sequencing could be obtained from the initial cultures, and tested if the cells could be expanded to evaluate growth and chemosensitivity under different culture conditions. In human normal keratinocytes, the Rho kinase (ROCK; ROCK1) inhibitor Y-27632 induces downregulation of genes involved in keratinization and differentiation (Chapman et al., 2014), while in malignant mouse mammary epithelial cells, use of CRC technology favors upregulation of keratin (*KRT*) differentiation genes when compared to mammary optimized media (Alamri et al., 2016). In mammary cells, this is associated with upregulation of tumor protein (TP) p53 family genes (Assefnia et al., 2014; Alamri et al., 2016). Therefore, the study compared expression of TP53 and salivary epithelial cell KRT differentiation-linked proteins (Azevedo et al., 2008; Zhu et al., 2015) in original tissue and cells cultured under CRC and non-CRC conditions.

RNA sequencing (RNAseq) is an established tool for unbiased transcriptome characterization and fusion gene screening (Nicorici et al., 2014; Tonella et al., 2017). Whole-exome sequencing is a means to evaluate DNA for known driver mutations, SNPs, and insertions and deletions (indels) (Nakagawa et al., 2015). RNAseq results from CRC cultured cells and original tissue show high concordance when passage number is limited (Alamri et al., 2016). Here, we tested whether combining CRC technology with next generation sequencing (NGS) could be used to correctly identify candidate therapeutic pathways present in the parenchyma of the original tissue.

## RESULTS

### Primary cultures from salivary gland neoplasms established using CRC technology were expanded under alternate culture conditions and yielded sufficient material at low passage numbers for NGS analyses

**Fig. 1.**
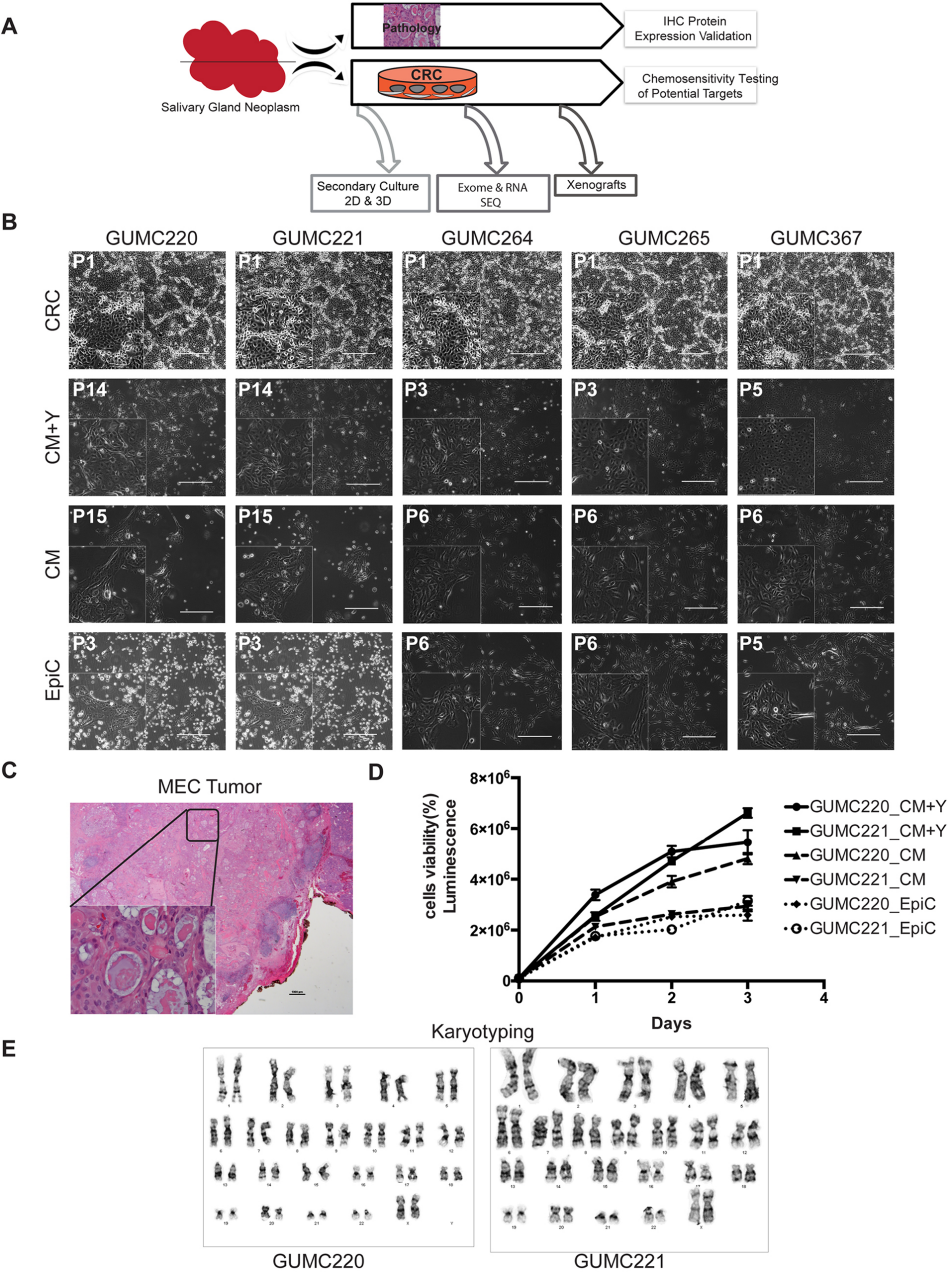
**Primary cancer cell cultures established in CRC were secondarily propagated without the presence of ROCK inhibitor or conditioned media.** (A) Overall experimental design. (B) Representative phase-contrast images of primary GUMC220/221 derived from a well-differentiated sublingual MEC in CRC, CM+Y, CM and EpiC media. GUMC264/265 and GUMC367 cultures derived from squamous cancers metastatic to parotid gland also grew in all three media. Passage numbers are indicated at the top left. Images were taken at 10× magnification. Scale bars: 400 μm. *n*=4 culture conditions per sample. GUMC220/221 and GUMC264/265 are biological replicate samples. (C) H&E-stained MEC tumor from which GUMC220 and GUMC221 biological replicate cultures were derived. Image was taken at 10× magnification. Scale bar: 10 μm. The bottom left inset shows a magnified image taken at 40× magnification. Scale bar: 10 μm. (D) GUMC220 and GUMC221 cell viability curves in CM+Y, CM, and EpiC media over 3 days. Each experiment included three cell/condition technical replicates and each experiment was completed three times. Luminescence: CellTiter-Glo^®^ (Promega). Data are mean±s.e.m. (E) Primary CRC-cultured GUMC220 and GUMC221 cells show normal karyotypes (46, XX). CRC, conditionally reprogramming cells; CM+Y, conditioned media+ROCK inhibitor (Y-27632); CM, conditioned media without Y-27632; EpiC, EpiCult™-C Human Medium. Karyotypes shown are representative of *n*=15 karyotypes evaluated for each.

**Fig. 2.**
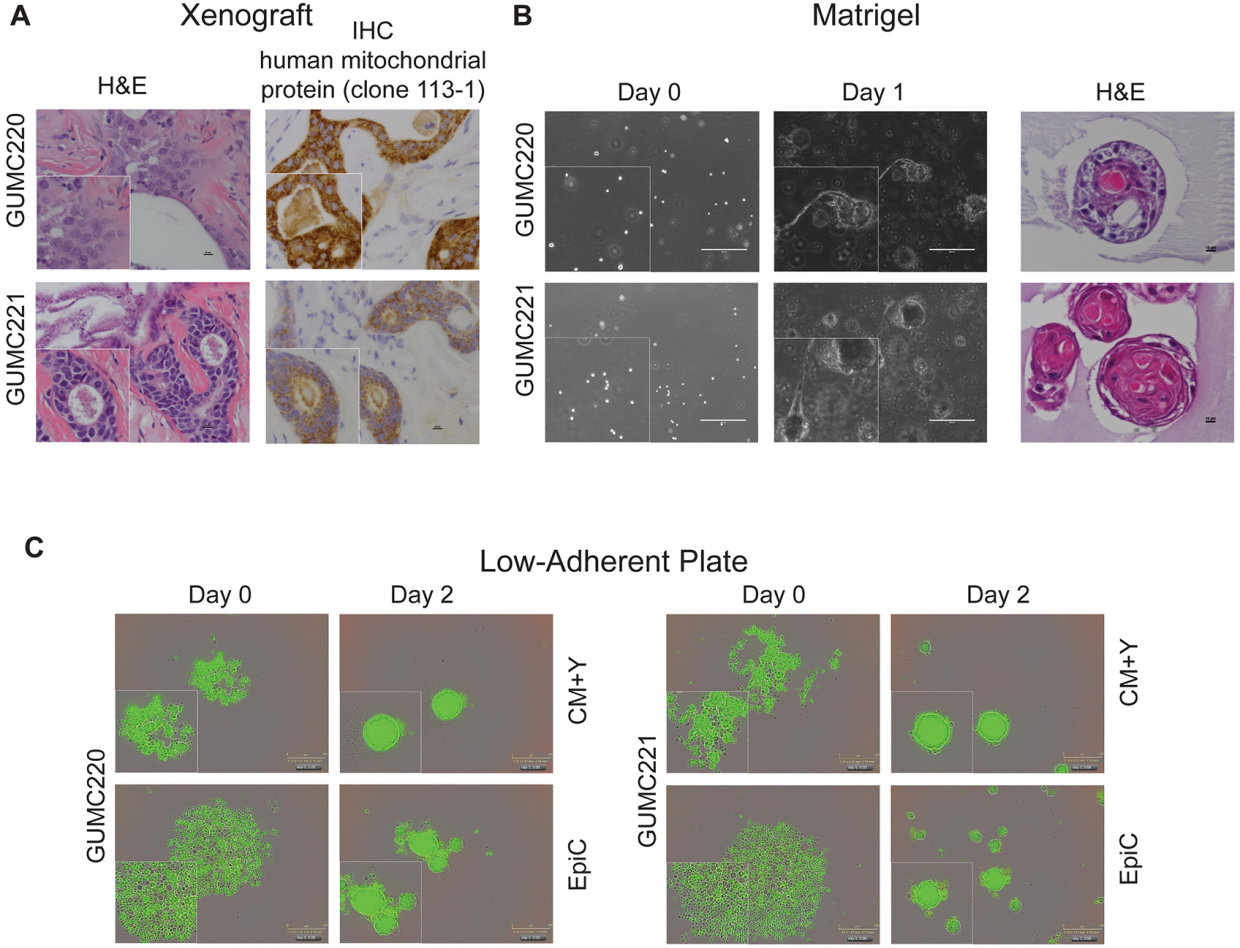
**GUMC220 and GUMC221** **MEC** **primary cells demonstrated xenograft growth *in vivo* and 3D growth *in vitro*.** (A) Representative images of xenograft histology: H&E (left), IHC for human mitochondrial protein (clone 113-1) (right). Images were taken at 40× magnification. Scale bars: 10 μm. Bottom left insets show magnified images; 220 xenografts, *n*=3; 221 xenografts, *n*=2. (B) Representative phase-contrast images from *in vitro* 3D Matrigel culture: single cell suspensions at day 0 (left), spheroid formation at day 7 (center), and representative H&E images of spheres (right). Images were taken at 10× magnification. Scale bars: 400 μm. Bottom left insets in day 0 and day 1 panels show magnified images. *n*=3. (C) Representative green fluorescence images from *in vitro* 3D low-adherent plate culture: single cell suspensions in CM+Y and EpiC at day 0 (left), spheroid formation in CM+Y and EpiC at day 2 (right). Images were taken at 10× magnification. Scale bars: 300 μm. Bottom left panels show magnified images. Each experiment included three cell/condition technical replicates and each experiment was completed three times.

Primary cultures from malignant MEC, carcinoma ex pleomorphic adenoma (ca ex PA), squamous cell carcinoma (SCC), squamous cell carcinoma metastatic to salivary gland (metastatic SCC), diffuse large B cell lymphoma in salivary gland, benign pleomorphic adenoma (PA), and benign ductal squamous metaplasia salivary gland neoplasms were established using CRC technology (Table S1). Specimens were obtained from surgically excised tissue with the exception of ca ex PA (attained from fine-needle aspirates). Paired independently derived cultures were established from different geographic regions of the same tumors (*n*=9) to be used for NGS and secondary culture studies (Fig. 1A). Formalin-fixed paraffin-embedded (FFPE) parent tissue was available for validation studies. Neither age nor sex influenced likelihood of culture establishment; however, no cultures could be established from the two sialadenitis cases attempted. The well-differentiated MEC [Georgetown University Medical Center (GUMC)220/221] and two metastatic to salivary gland SCC specimens (GUMC264/265 and GUMC367) were selected for secondary culture studies (Table S2). All cells expanded for multiple passages (3-15) under the three secondary 2D culture conditions tested (Fig. 1B), including the well-differentiated low-grade MEC (Fig. 1C). Paired cultures demonstrated similar secondary culture kinetics but expansion was fastest using conditioned medium (CM)+Y-27632 (Y) (Fig. 1D). Karyotype was normal for the GUMC220/221 cells grown in CRC (Fig. 1E). While all five cultures propagated in alternative media, including EpiCult™-C (EpiC) (StemCell Technologies, Vancouver, CN), only GUMC220/221 formed xenografts (Fig. 2A; Table S2). GUMC220/221 also propagated in MammoCult™ (StemCell Technologies) and formed spheroids when grown in Matrigel and on low-adherent plates with both CM+Y and EpiC media (Fig. 2B,C). Sufficient cells for NGS were obtained after two (GUMC220/221, GUMC264/265, GUMC299, GUMC367, GUMC332/349, GUMC311, GUMC436/446, GUMC572/573), three (GUMC374/378, GUMC312) and four (GUMC284) passages. The magnitudes of statistically significant differentially expressed genes (DEGs) were <0.4% of genes expressed for all seven paired cultures examined (range 0-0.38%) (Table S3). Hierarchical clustering demonstrated limited dissimilarity between paired specimens (Fig. S1), but all cultures demonstrated significant expression of *KRT* genes consistent with epithelial origin. No known pathogenic cancer mutations or driver genes were identified; however, cancer risk-related probable pathogenic SNPs were reproducibly present in both of the four paired samples subjected to exome sequencing (Table S4; Agalliu et al., 2010; Alvarez-Cubero et al., 2016; Cheng et al., 2014; Chou et al., 2017; Cui et al., 2016; Dai et al., 2014; Dong et al., 2016; Ewart-Toland et al., 2005; Guo et al., 2014; Hu et al., 2014; Huang and Yang, 2015; Jiao et al., 2012; Johnson et al., 2007; Tanaka et al., 2010; Tian et al., 2017; Xu et al., 2015; Yan et al., 2016). In GUMC220/221, these included *Brca2 rs144848*, *TP53* rs1042522, *AURKA* rs2273535, *RET* rs1800858 and *ADH1B* rs1229984.

### An activated *AREG-EGFR-AKT* pathway but no *CRTC1-MAML2* fusion gene was identified in the well-differentiated MEC by transcriptome analyses

**Fig. 3.**
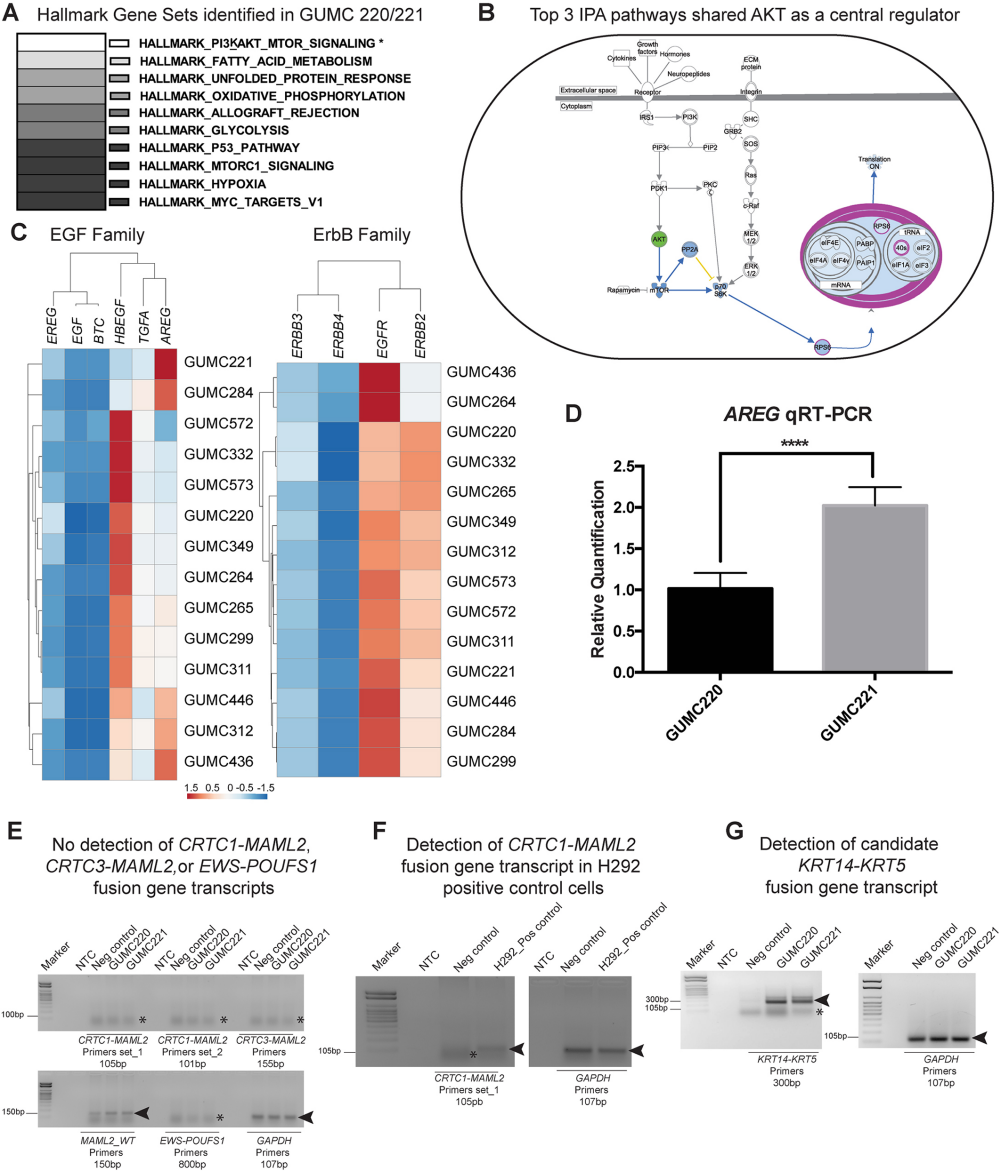
**Transcriptome characterization of GUMC220 and GUMC221 MEC primary cells demonstrated evidence of PI3K**-**AKT-mTOR­** **signaling activation and presence of a candidate KRT14-KRT5 fusion.** (A) Top 10 Hallmark gene sets (MSigDB) identified from genes showing ≥500 FPKM. The top 10 pathways were identical in GUMC220 and GUMC221. Only PI3K-AKT-mTOR signaling was significantly associated with GUMC220/221 compared to the six other paired culture transcriptomes analyzed (four pleomorphic adenomas: GUMC284/299, GUMC332/349, GUMC436/446, GUMC572/573; one metastatic squamous to parotid: GUMC264/265; one submandibular lymphoma: GUMC311/312). *P<0.05, two-tailed Fisher’s exact test. (B) Top three canonical pathways identified by IPA shared AKT as a central regulator. (C) Dendrograms and heat maps of unsupervised hierarchical clustering of FPKM values of genes from EGF (*EREG*, *EGF*, *BTC*, *HBEGF*, *TGFA*, *AREG*) and ErbB (*ERBB3*, *ERBB4*, *EGFR*, *ERRB2*) families. (D) *AREG* expression in GUMC220 and GUMC221 cells, evaluated by qRT-PCR. ****P<0.0001, two-tailed Student's *t*-test, *n*=3. Data are mean±s.e.m. (E) Ethidium bromide-stained agarose gels showing absence of detection of *CRTC1-MAML2*, *CRTC3-MAML2* and *EWS-*POUFS1 fusions (asterisks indicate primer dimers) and detection of *MAML2-WT* and *GAPDH* (arrowheads) by RT-PCR in GUMC220 and GUMC221 cells. Predicted product sizes are included under each primer label. (F) Ethidium bromide-stained agarose gels showing detection of *CRTC1-MAML2* in H292 cell line as a positive control (arrowheads). Asterisks indicate primer dimers. Predicted product sizes are included under each primer label. (G) Ethidium bromide-stained agarose gels showing detection of candidate *KRT14-KRT5* fusion and *GAPDH* (arrowheads) in GUMC220 and GUMC221 cells. Asterisks indicate primer dimers. *AREG*, amphiregulin; AKT, protein kinase B; *BTC*, betacellulin; *CRTC1*, CREB regulated transcription coactivator 1; *CRTC3*, CREB-regulated transcription coactivator 3; *EGFR*, epidermal growth factor receptor; *ERBB2*, Erb-B2 receptor tyrosine kinase 2; *ERBB3*, Erb-B2 receptor tyrosine kinase 3; *ERBB4*, Erb-B2 receptor tyrosine kinase 4; *EREG*, epiregulin; PI3K, phosphatidylinositol-4,5-bisphosphate 3-kinase; *EWS*, Ewing's sarcoma gene; EGF, epidermal growth factor; *HBEGF*, heparin binding EGF-like growth factor; *MAML2*, mastermind-like transcriptional coactivator 2; mTOR, mechanistic target of rapamycin; Neg control, negative control (GUMC-UMB-006 cells isolated using CRC from normal human tongue); NTC, no template control; Pos control, positive control; *POUFS1*, POU class 1 homeobox 1; *TGFA*, transforming growth factor alpha.

To identify cancer-associated signaling pathways amenable to *in vitro* therapeutic testing, the Molecular Signatures Database (MSigDB) v6.0 (http://software.broadinstitute.org/gsea/msigdb) was interrogated to identify the top 10 Hallmark gene sets with significant overlap with genes expressing ≥500 fragments per kilobase of transcript per million mapped reads (FPKM) for each paired culture. The PI3K-AKT-mTOR signaling Hallmark gene set was uniquely associated with MEC cultures GUMC220/221 (*P*<0.05, Fisher’s exact, Fig. 3A). In addition, the top three canonical pathways identified from Ingenuity Pathway Analysis (IPA) of GUMC220/221 (genes ≥500 FPKM) shared AKT as a central regulator [eukaryotic translation initiation factor (EIF) signaling, regulation of EIF-4 and ribosomal protein S6 kinase (p70Sk) signaling, mTOR signaling] (Fig. 3B). Comparison of *EGF* and *ErbB* family member FPKM levels demonstrated that *AREG*, heparin binding EGF-like growth factor (*HBEGF*), *EGFR* and *ERBB2* were expressed at relatively higher levels than other family members in all paired samples (Fig. 3C). GUMC220/221 demonstrated differential *AREG* expression levels with statistically significantly higher levels in GUMC221 as compared to GUMC220 on both bioinformatics analysis (Trapnell et al., 2012, 2010) and quantitative reverse transcriptase-polymerase chain reaction (RT-PCR) analysis (Fig. 3D). Neither a bioinformatics approach (FusionCatcher, Nicorici et al., 2014) nor RT-PCR using established primer sets identified any known MEC-associated fusion gene transcripts (Fig. 3E,F; Table S5). A candidate novel fusion gene (*KRT14-KRT5*) was identified using FusionCatcher (Table S6). Primers designed around the predicted fusion site revealed a product of the expected size on RT-PCR (Fig. 3G; Table S7, Fig. S2A). Sequencing of the PCR product revealed a candidate junction site (Fig. S2B) with the predicted location of the candidate fusion within exon 1 (Fig. S2C). Fluorescence *in situ* hybridization (FISH) was performed on cultured cancer cells and the original mucoepidermoid cancer to determine whether K14 and K5 probes would colocalize to the same chromosome, but results were not definitive (Fig. S2D,E).

### An activated AREG-EGFR-AKT pathway was confirmed as present in the original tissue and xenograft of the well-differentiated MEC

**Fig. 4.**
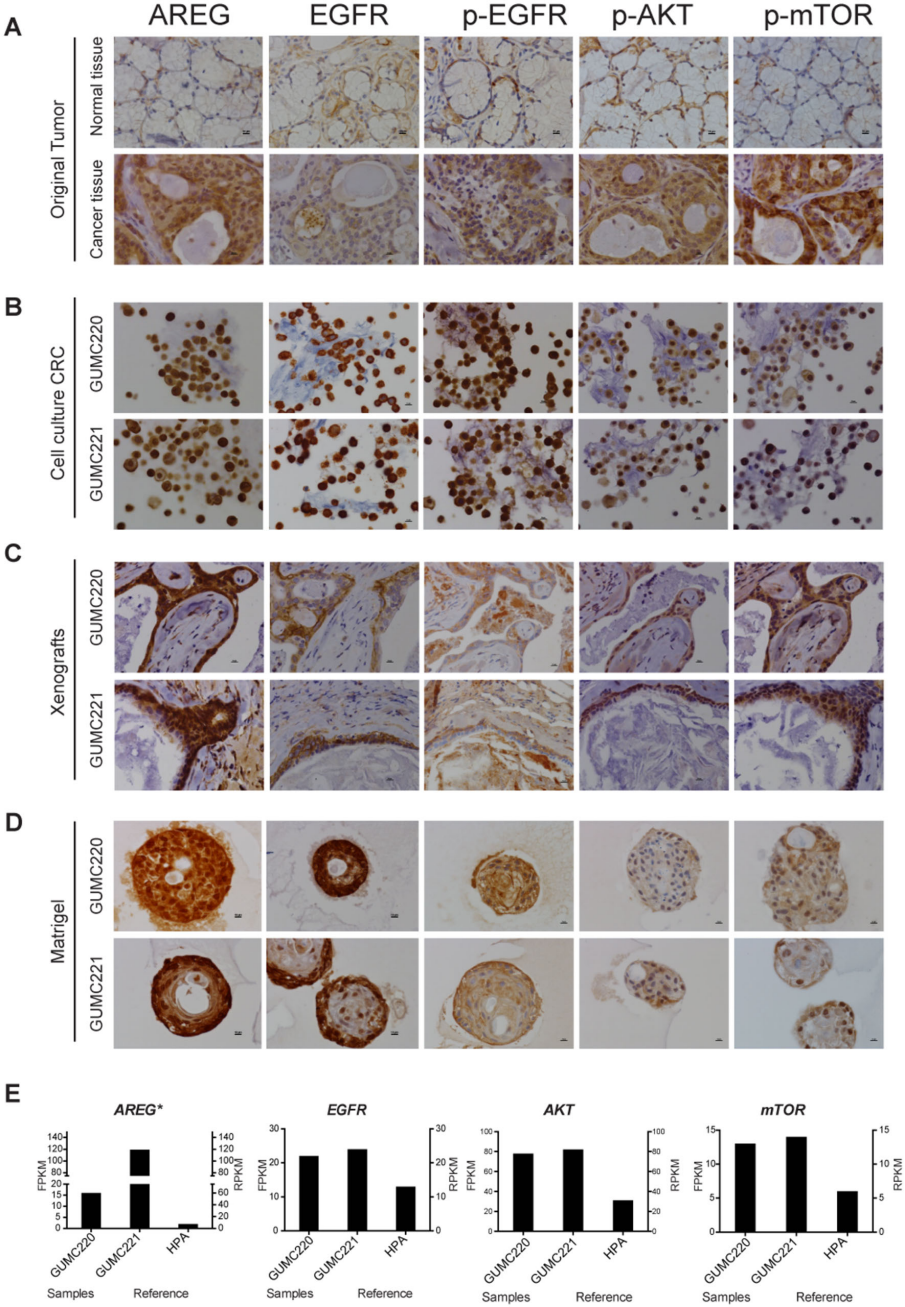
**Evaluation of AREG, EGFR, p-EGFR, p-AKT and p-mTOR expression in original tumor and GUMC220/221 CRC cultures, xenografts and Matrigel cultures.** (A-D) Representative IHC images for AREG, EGFR, p-EGFR, p-AKT and p-mTOR in adjacent normal tissue (top) and MEC (cancer tissue, bottom) (A), GUMC220 (top) and GUMC221 (bottom) cell pellets from CRC cultures (B), GUMC220 (top) and GUMC221 (bottom) xenografts (C), and GUMC220 (top) and GUMC221 (bottom) Matrigel spheroids (D). Images were taken at 40× magnification. Scale bars: 10 μm. (E) Left *y*-axis: relative FPKM values for *AREG*, *EGFR*, *AKT* and *mTOR* in GUMC220 and GUMC221. *P<0.05 (Cuffdiff). Right *y*-axis: for reference, relative RPKM reads for *AREG*, *EGFR*, *AKT* and *mTOR* from the HPA for normal salivary gland tissue.

**Fig. 5.**
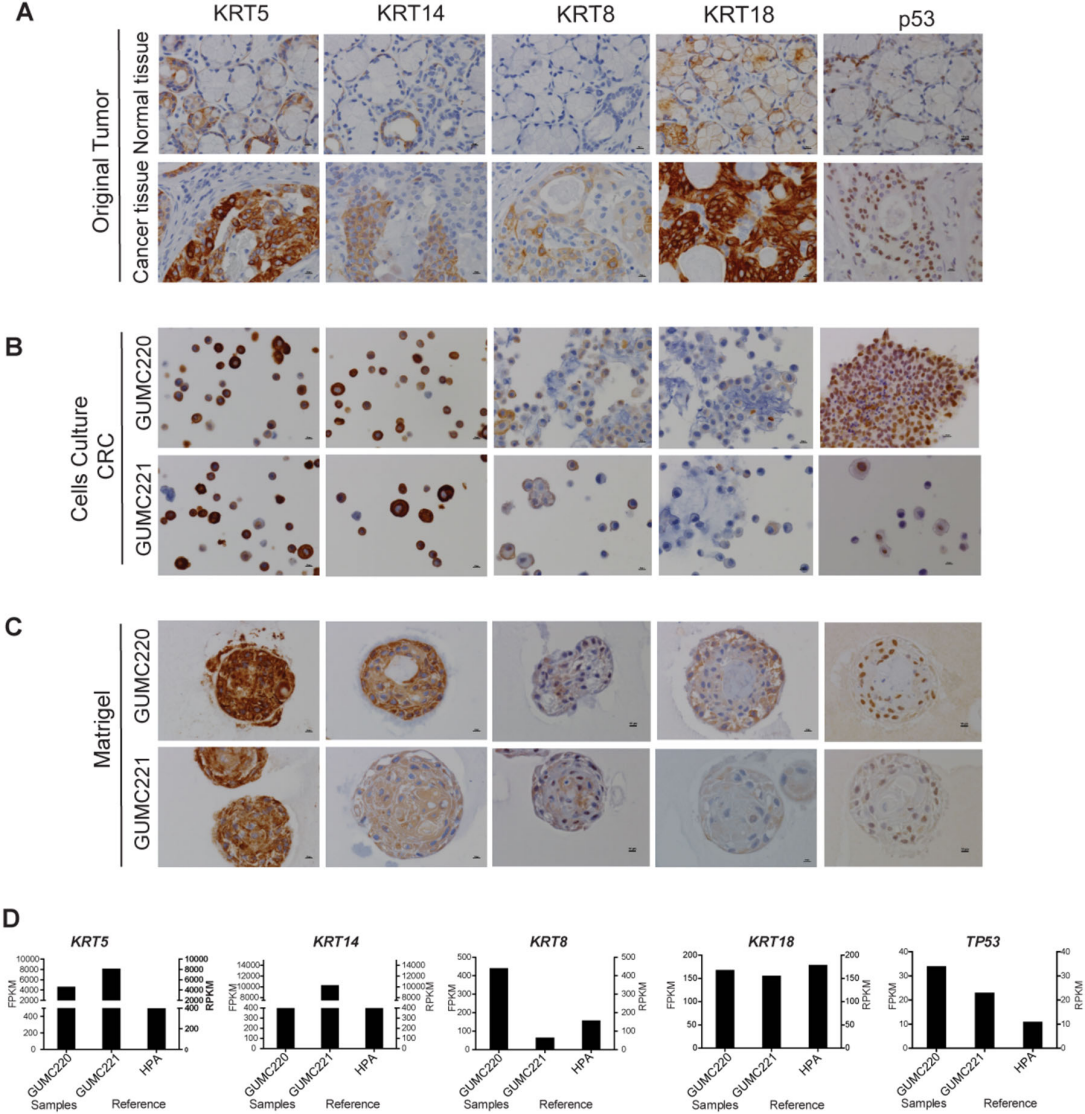
**Evaluation of KRT5, KRT14, KRT8, KRT18 and p53 in original tumor and GUMC220/221 CRC and Matrigel cultures.** (A-C) Representative IHC images for KRT5, KRT14, KRT8, KRT18 and p53 in adjacent normal tissue (top) and MEC (cancer tissue, bottom) (A), GUMC220 (top) and GUMC221 (bottom) cell pellets from CRC cultures (B), and GUMC220 (top) and GUMC221 (bottom) Matrigel spheroids (C). Images were taken at 40× magnification. Scale bars: 10 μm. (D) Left *y*-axis: relative FPKM values for *KRT5*, *KRT14*, *KRT18* and *Tp53* in GUMC220 and GUMC221. Right *y*-axis: for reference, relative RPKM reads for *KRT5*, *KRT14*, *KRT18* and *Tp53* from the HPA for normal salivary gland tissue.

Presence of AREG, p-EGFR, p-AKT and p-mTOR was confirmed in the cancer tissue, CRC cells and xenografts by immunohistochemistry (IHC) (Fig. 4A-C). However, although Matrigel cultures showed preservation of AREG and EGFR expression, reactivity for p-AKT and p-mTOR were lower compared to other specimens (Fig. 4D). Reads per kilobase of transcript per million mapped reads (RPKM) reported for normal salivary gland from the Human Protein Atlas (HPA; https://www.proteinatlas.org/) with FPKM levels for *AREG*, *EGFR*, *AKT* and *mTOR* from MEC GUMC220/221 are shown for comparison (Fig. 4E). KRT protein expression was evaluated for concordance between original tissue and cultured cells to follow up on the candidate *KRT14-KRT5* fusion gene identified in cultured cells. KRT8, KRT18 and TP53 were included for comparison because they are also expressed at relatively higher levels under CRC conditions (Alamri et al., 2016). Reactivity for KRT5/18 was higher than that for KRT14/8 in the original MEC (Fig. 5A). By contrast, primary cells in CRC showed higher reactivity for KRT5/14 than for KRT8/18, and this pattern was largely preserved in Matrigel culture (Fig. 5B,C). Nuclear-localized TP53 expression found in the original cancer was maintained in the CRC and Matrigel cultures (Fig. 5A-C). RPKM reported for normal salivary gland from the HPA with FPKM levels for *KRT5/14/8/18* and *TP53* from MEC GUMC220/221 are shown for comparison (Fig. 5D).

### MEC GUMC220/221 cells were significantly more sensitive to AKT inhibitor MK-2206 than to GSK690693

**Fig. 6.**
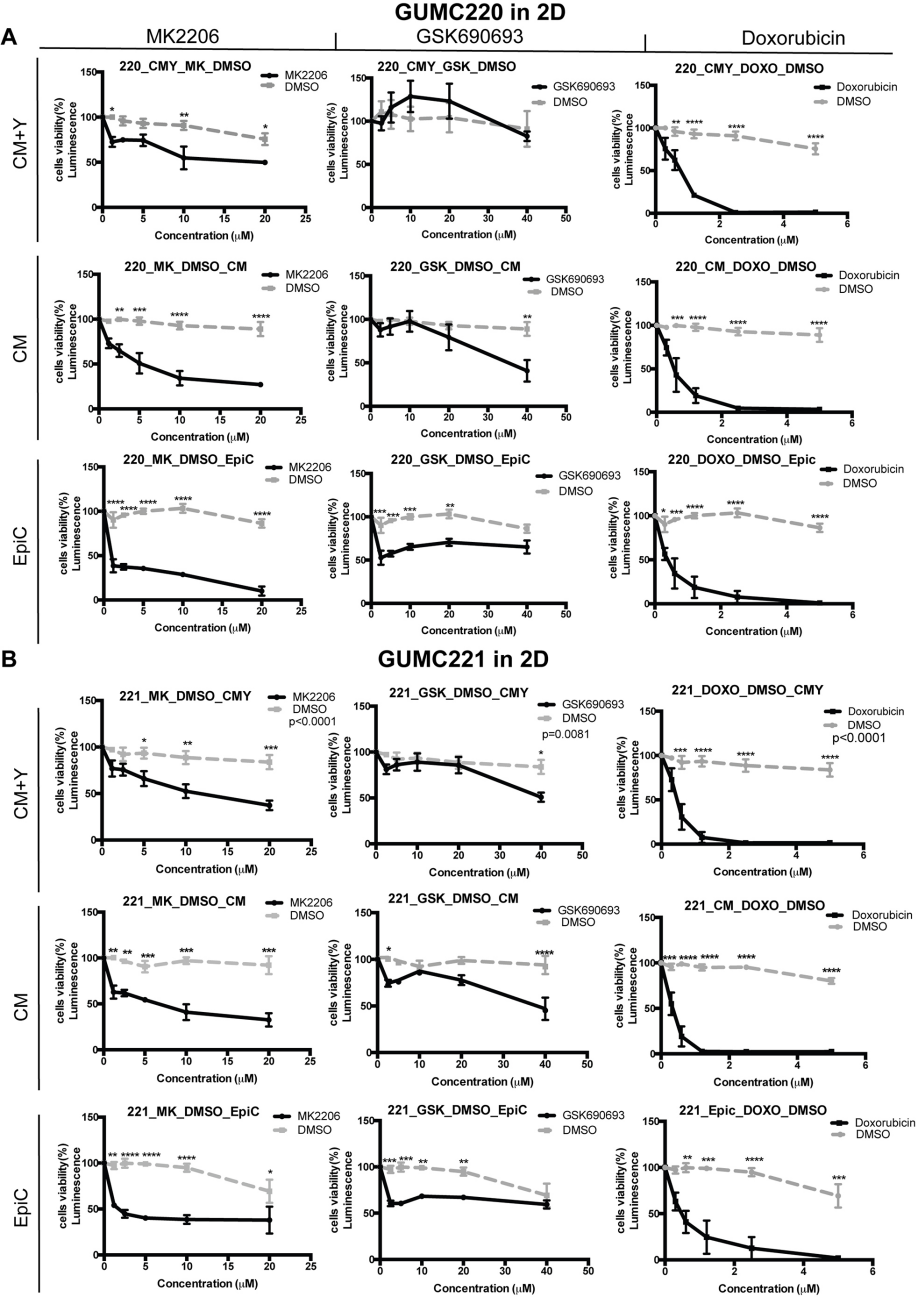
**Viability of GUMC220/221 2D cell cultures exposed to MK2206, GSK690639 and doxorubicin in CM+Y, CM and EpiC media.** (A,B) Cell viability measured after 3 days for GUMC220 (A) and GUMC221 (B) in three different 2D culture media (CM+Y, CM and EpiC), at a range of concentrations in the presence of MK2206 (1.2-20 μM, solid black line) compared to DMSO (vehicle control, dashed gray line) (left column), GSK690639 (2.5-40 μM, solid black line) compared to DMSO (vehicle control, dashed gray line) (middle column), and doxorubicin (0.3-5 μM, solid black line) compared to DMSO (vehicle control, dashed gray line) (right column). Data are mean±s.e.m. One DMSO vehicle control was used for each medium each time an experiment was performed (dashed gray line). Each experiment included three technical replicates, and each experiment was performed three times. *P≤0.05, **P≤0.01, ***P≤0.001, ****P≤0.0001, one-tailed, one-way ANOVA.

**Fig. 7.**
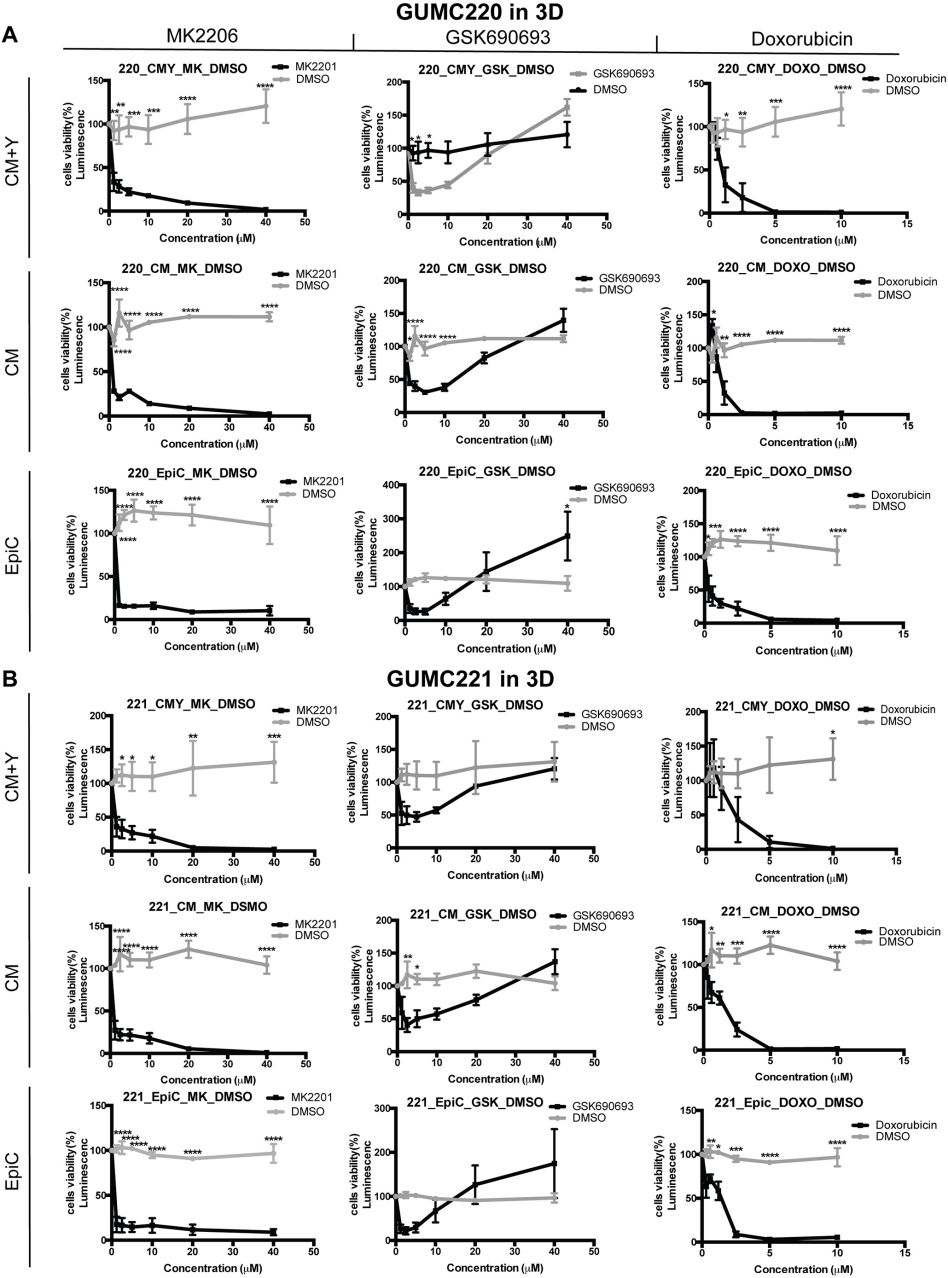
**Viability of GUMC220/221 3D cell cultures exposed to MK2206, GSK690639 and doxorubicin in CM+Y, CM and EpiC media.** (A,B) Cell viability measured after 3 days for GUMC220 (A) and GUMC221 (B) in three different 3D culture media (CM+Y, CM and EpiC) at a range of concentrations in the presence of MK2206 (1.2-40 μM, black line) compared to DMSO (vehicle control, gray line) (left column), GSK690639 (2.5-40 μM, black line) compared to DMSO (vehicle control, gray line) (middle column), and doxorubicin (0.6-10 μM, black line) compared to DMSO (vehicle control, gray line) (right column). Data are mean±s.e.m. One DMSO vehicle control was used for each medium each time an experiment was performed (gray line). Each experiment included three technical replicates, and each experiment was performed three times. *P≤0.05, **P≤0.01, ***P≤0.001, ****P≤0.0001, one-tailed, one-way ANOVA.

**Fig. 8.**
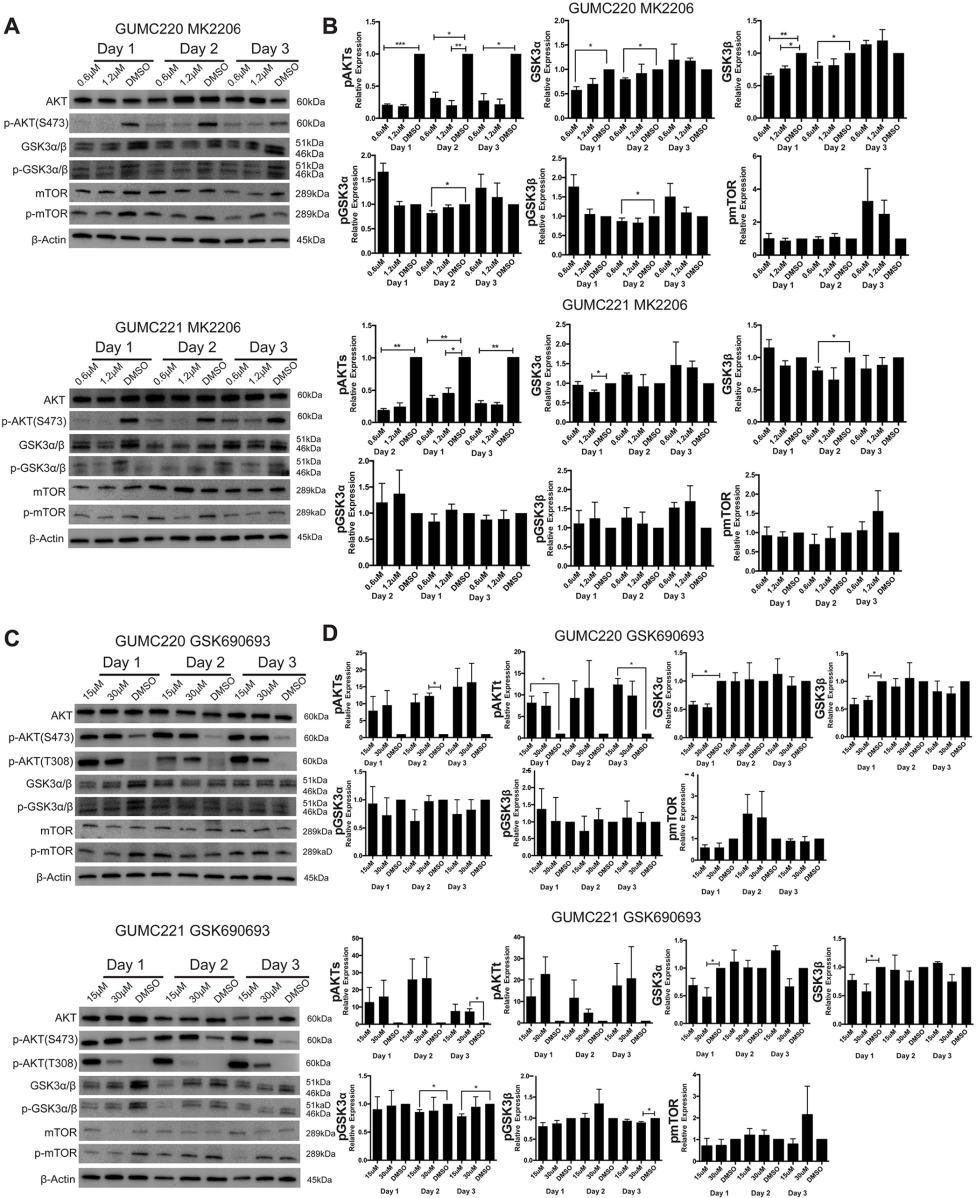
**Western blot analyses of steady-state levels of AKT, *P*-AKT, GSK3α/β, p-GSK3α/β, mTOR and p-mTOR following exposure to MK2206 and GSK690693 in 2D culture (CM+Y).** (A) Representative western blots of AKT, p-AKT(S473), GSK3α/β, p-GSK3α/β, mTOR, p-mTOR and β-actin from GUMC220 and GUMC221 exposed to MK2206 (0.6 μM and 1.2 µM) compared with DMSO vehicle control. (B) Steady-state expression levels of pAKT(S473), GSK3α/β, p-GSK3α/β and p-mTOR in GUMC220 and GUMC221 cells after 1, 2 and 3 days’ exposure to the two different concentrations of MK2206 and DMSO. Data are mean±s.e.m., *n*=3/timepoint/concentration. (C) Representative western blots of AKT, p-AKT(S473), p-AKT(T308), GSK3α/β, p-GSK3α/β, mTOR, p-mTOR and β-actin from GUMC220 and GUMC221 in 2D CM+Y culture exposed to GSK690693 (15 μM and 30 µM) compared with DMSO vehicle control. (D) Steady-state expression levels of pAKT(S473), p-AKT(T308), GSK3α/β, p-GSK3α/β and p-mTOR in GUMC220 and GUMC221 cells after 1, 2 and 3 days’ exposure to the two different concentrations of GSK690693 compared with DMSO vehicle control. Phosphoprotein levels were normalized to total proteins, which were normalized to actin. *P≤0.05, **P≤0.01, ***P≤0.001, one tailed, unpaired *t*-test. Protein lysates were collected after 1, 2 and 3 days of drug exposure. Data are mean±s.e.m., *n*=3/timepoint/concentration. Molecular weights (kDa) are indicated for each protein.

Survival of GUMC220/221 primary cells was assessed following exposure to AKT inhibitors MK2206 and GSK690693 over a range of concentrations in 2D and 3D culture conditions using the three different media shown to propagate the cells (CM+Y, CM and EpiC). Doxorubicin was used as a comparative control for the two AKT inhibitors. MDA-MD-453 cells were used as a positive control for GSK690693 (Kumar et al., 2010). Survival was statistically significantly and reproducibly reduced under all conditions by MK-2206 as well as doxorubicin but not GSK690693 (Figs 6 and 7). By contrast, survival of MDA-MD-453 cells was reproducibly reduced in all three media (Fig. S3A). Western blot analyses were performed to determine whether the drugs induced expected changes in p-AKT, p-GSK3α/β and p-mTOR. Steady-state protein levels were compared after 1, 2 and 3 days of exposure to MK2206, GSK690693 and DMSO vehicle control (Fig. 8). p-AKT levels were reproducibly reduced after exposure to MK2206 and increased after exposure to GSK690693; however, consistent changes in p-GSK3α/β and p-mTOR were not found. By contrast, positive control MDA-MD-453 cells demonstrated the expected increase in p-AKT and reductions in p-GSK3α/β and p-MTOR following GSK690693 exposure (Fig. S3B). Because ERBB2 (HER2) overexpression has been linked to the differential pattern of MK2206 and GSK690693 sensitivity found here (Vivanco et al., 2014), RNA and protein expression levels were evaluated in GUMC220/221 but no increases were found (Fig. 3C; Fig. S4). Because reduced *AKT3* expression levels in triple-negative breast cancers are reported to increase sensitivity to GSK690693 (Chin et al., 2014), FPKM values of *AKT1/2/3* were checked in GUMC220/221. FPKM levels were highest for *AKT1* (82.28/77.81) followed by *AKT2* (27.25/22.38) and *AKT3* (1.17/1.62).

## DISCUSSION

Primary cell cultures were established from all of the benign, primary cancer and metastatic samples tested, although not the two chronic sialadenitis cases. Chronic sialadenitis is an inflammatory disease that leads to atrophy and loss of acinar structures (Wilson et al., 2014). It is possible that either or both the presence of inflammation or diminished epithelia contributed to the lack of success with this disease. Epithelial cells were isolated from a salivary gland containing a lymphoma (GUMC311/312), demonstrating an approach for isolating nonmalignant epithelial cells from the lymphoma environment for studies targeted towards the study of heterogeneous cell populations in malignancy. MEC GUMC220/221 cultures were able to form a patient-derived xenograft but this was not true of all cultures attempted. The same has been reported for CRC-derived cells from human pancreatic cancers (Beglyarova et al., 2016). It could be notable that only the MEC GUMC220/221 cells carried a cancer risk-associated *TP53* SNP altering p53 function (Pim and Banks, 2004). CRC-derived murine cancer cells with *Trp53* haploinsufficiency effectively form allografts (Alamri et al., 2016).

The primary human cultures established using the classic CRC technology were secondarily propagated in alternative media, as reported previously for murine cells (Alamri et al., 2016). They also propagated in 3D culture formats. 3D culture formats have been proposed as being more representative of the *in vivo* setting leading to more accurate predictive chemosensitivity testing (Unger et al., 2015). Here, the differential sensitivity of the two AKT inhibitors was present in both 2D and 3D culture formats. It also was present across the different culture media tested. Because Rho kinases are linked to multiple roles in carcinogenesis (Wei et al., 2016) and Rho kinase inhibitors impact cellular physiology in several ways (Liao et al., 2007; Feng et al., 2016), a concern was whether or not the presence of Y-27632 would distort chemotherapeutic testing. But this was not the case for the agents tested – MK2206, GSK690693 and doxorubicin – as was previously shown for Vorinostat tested in CRC cells from an HPV11-related laryngeal carcinoma (Yuan et al., 2012).

The differential response to the two different AKT inhibitors underscores the more general need for a further understanding of the genetic settings in which specific anticancer drugs will be active. The relative resistance to GSK690693 in the MEC cells appeared to be innate rather than acquired in culture as it was evident on first testing and found in all culture formats. Significantly, previously described mechanisms of GSK690693 resistance were not present. In the clinic, AKT inhibitors are anticipated to be used in combination with other chemotherapeutic agents, not as single agents (Nitulescu et al., 2016). One next step would be to evaluate response to MK2206 in rational combinations with other agents active in salivary gland cancers (Chintakuntlawar et al., 2016). More generally, the approach described here can be used to expand testing of human primary cancer cells to further our knowledge of chemosensitivity of salivary gland cancers.

Reproducible results from RNAseq and exome sequencing were obtained from cells established in CRC at low passage, providing data specifically on the epithelial component of the tissue and mitigating variables associated with more prolonged cell culture including gene expression drift (Alamri et al., 2016). There was high concordance on genetic findings from each of the paired samples, even though they were derived from geographically distinct sites within the tumors. This could indicate that single specimens might be sufficient. At the same time, the high levels of AREG expression found in the MEC reported here were only present in one of the two primary cultures, perhaps arguing against single specimens for precision medicine.

As compared to sequencing a collection of primary cells, which provides information on a population of cells, single cell sequencing (SCS) can be used to study individual cells within a population and explore cell heterogeneity, and has been used as an approach to investigate tumor evolution (Tang et al., 2009; Navin et al., 2011; Wang and Navin, 2015). Circulating cancer cells have been studied using SCS to define candidate driver mutations in primary and metastatic lesions (Heitzer et al., 2013; Lohr et al., 2014). SCS is a technology that continues to be improved as possible technical errors can contribute to identification of heterogeneity that is not actually present. Population sequencing as employed here is more economical for obtaining information on thousands of cells, still provides the possibility for detection of rare events, and enables the use of sequenced cells in functional assays as performed here (Wang and Navin, 2015). It is possible that CRC technology could be used in combination with SCS as low numbers of cells can be cultured with this methodology, as illustrated in this study, by the culture of the fine-needle aspirate samples.

In salivary gland cancers, fusion genes are seen as key molecular drivers (Stenman et al., 2014). In MEC, they are reported to be more frequently present in low-grade MEC and in association with AREG overexpression, but that was not the case for the MEC studied here. The presence of a novel *KRT14-KRT5* fusion gene here could not be fully confirmed at the DNA level. FPKM values for *KRT14* and *KRT5* were relatively high in the cultured cells. It is possible the fusion detected could be a chimeric RNA or a spurious finding, as has been previously reported for RNAseq data (Jividen and Li, 2014; Peng et al., 2015).

In summary, the approach employed here can reasonably, rapidly and cost-effectively expand primary cell cultures from salivary gland neoplasms for genetic and chemosensitivity studies, extending our understanding of the pathophysiology of these relatively uncommon cancer types and providing a foundation for precision medicine.

## MATERIALS AND METHODS

### Primary salivary gland epithelial cell culture, xenograft and cytogenetic analysis

Fresh tissue not required for pathological diagnosis was serially collected over approximately 1 year from consenting patients with salivary gland neoplasms. Informed consent was obtained from all patients in compliance with relevant ethical regulations by the Non-Therapeutic Subject Registry (NTSR) working under Georgetown University Institutional Review Board approval, and all clinical investigation was conducted according to the principles expressed in the Declaration of Helsinki. Tissue obtained from surgically excised specimens (0.5-1 cm^3^ specimen; *n*=8 paired samples, *n*=3 single specimens) or fine-needle aspirates (*n*=1 paired sample) was provided to the Histopathology and Tissue Shared Resource (HTSR), where identifiers were removed and unique codes applied before transfer to the Tissue Culture Shared Resource (TCSR) for primary culture. Two specimens from different areas of the pathological samples were provided when sufficient tissue was available and each processed as individual specimens for comparison (*n*=9 paired samples). Paired samples were authenticated as derived from the same tissue by their patterns of SNPs. No fungal or bacterial contamination of cultures was identified during the study period. Deidentified pathology reports were provided by the HTSR for all samples. Primary cells were isolated using 1× collagenase/hyaluronidase solution (ThermoFisher Scientific, Waltham, MA) followed by 0.25% trypsin-EDTA (ThermoFisher Scientific) from fresh tissue (surgical specimens) or placed directly into culture without processing (fine-needle aspirates) in complete F medium [Ham's F-12 nutrient mix (25%) (ThermoFisher Scientific) supplemented with 25 ng/ml hydrocortisone, 5 μg/ml insulin, 0.1 nmol/l cholera toxin (Sigma-Aldrich, St Louis, MO), 0.125 ng/ml epidermal growth factor, 10 μg/ml gentamicin (ThermoFisher Scientific), 250 ng/ml Fungizone (ThermoFisher Scientific), 5 μmol/l ROCK inhibitor Y-27632 (Y) (Enzo Life Sciences, Farmingdale, NY)] and 74% complete Dulbecco's modified Eagle's medium (DMEM) [10% fetal bovine serum (FBS), 100 μg/ml penicillin, 100 μg/ml streptomycin, 100 μg/ml glutamine (ThermoFisher Scientific)] in the presence of irradiated Swiss 3T3-J2 mouse fibroblast feeder cells at 37°C with 5% CO_2_ (Chapman et al., 2010; Liu et al., 2017, 2012), and assigned a unique Georgetown University Medical Center (GUMC) primary cell culture identifier. For secondary passage, primary cells were separated from feeder layers by differential trypsin treatment (30 s, 0.05% trypsin, ThermoFisher Scientific) followed by wash with 1× phosphate buffered saline (PBS) to remove detached feeder layers, and subjected to a second treatment with 0.05% trypsin to detach epithelial cells before placement in conditioned medium+5 μmol/l Y (CM+Y), conditioned medium without Y-27632 (CM), EpiC [EpiCult™-C Human Medium Kit containing 5 ml EpiCult™-C Proliferation Supplement (Human), hydrocortisone (10^−6^ M)] (Stemcell Technologies, Vancouver, BC), 100 μg/ml glutamine and 100 μg/ml streptomycin/penicillin (ThermoFisher Scientific), or MammoCult™ Human Medium Kit supplemented with MammoCult™ Proliferation Supplement (Human) 50 ml, hydrocortisone (10^−6^ M), 2 μg/ml heparin solution (Stemcell Technologies, Vancouver, BC), and 100 μg/ml streptomycin/penicillin (ThermoFisher Scientific). Conditioned medium was prepared by plating irradiated Swiss 3T3-J2 mouse fibroblast feeder cells (7×10^6^ /T175 cm^2^ tissue culture flask) in complete F medium with collection of supernatant F medium (CM), followed by filtration through 0.22-mm pore-size filter (EMD Millipore, Billerica, MA) after 3 days and storage at −80°C. Before use, it was mixed with complete F medium (75% CM/25% complete F) (Palechor-Ceron et al., 2013). Primary cell cultures were viably frozen and recultured when needed. For 3D, embedded cultures in Matrigel (BD Biosciences, Franklin Lakes, NJ), cells were grown to 80-90% confluency, detached by trypsinization, resuspended in complete F medium+Y (4×10^4^ cells/suspension) on ice, mixed with 1.2 ml Matrigel, plated on the surface of precoated six-well culture plates with 2 ml F medium+Y added on top, and incubated at 37°C with 5% CO_2_ (Lee et al., 2007). To precoat plates, prechilled six-well culture plates were coated with a thin layer of Matrigel (200 µl) and incubated at 5 min at 37°C. Cultures were maintained for 10 days. For 3D spheroid culture, 1.5×10^4^ cell/100 µl of CM+Y, CM and EpiC was plated in 96-well round-bottom low-attachment plates (Corning, NY), centrifuged at 234 ***g*** (5 min), and incubated at 37°C with 5% CO_2_. Vybrant™ Cell-Labeling Solution (ThermoFisher Scientific) was used to label cells with green fluorescence (10 µl cell-labeling solution/1 ml cell suspension) with incubation at 37°C (30 min), followed by centrifugation at 5 ***g*** (5 min), removal of the supernatant, washing three times in warm medium and resuspension. Digital images of 2D and 3D primary cell cultures were taken using an EVOS™ XL Core Cell Imaging System (ThermoFisher Scientific). For xenograft development, 6-week-old athymic nude [*NU (NCr)-Foxn1nu*] female mice (*n*=20) (Harlan Laboratories, Inc., Frederick, MD) (26-30 gm) were housed in barrier zones in single-sex sterilized ventilated cages at Georgetown University and acclimatized 1 week prior to primary cell inoculation [10^6^/site in 25 μl PBS/25 μl Matrigel (BD Biosciences, Franklin Lakes, NJ) via a 1-cm^3^ syringe/27-gauge needle] into thoracic and inguinal mammary fatpads (four sites/mouse) and into the flank subcutaneously (two sites/mouse) through a skin incision under isoflurane anesthesia, performed by an investigator (W.W.) blinded to the identity of the cell cultures. Mice were monitored weekly with measurement of palpable and visible tumors, and euthanized by CO_2_ inhalation followed by cervical dislocation when mice reached 6 months of age or palpable tumors >1 cm^3^, whichever occurred first. At necropsy, mice were examined for xenograft growth at injected sites, and tissue removed for formalin fixation and processing for pathological examination. All animal procedures were performed and approved by the GUMC Institutional Animal Care and Use Committee. For conventional cytogenetic analysis (karyotyping), primary GUMC220/221 cells were cultured in F media+Y [passage (P) 9 for both]. Chromosome preparation and G-banding assays were performed using standard protocols (Arsham et al., 2017), and chromosomes identified and classified according to standard cytogenetic nomenclature (McGowan-Jordan et al., 2016).

### RNAseq, exome sequencing and analyses

Primary cells cultured in CRC were pelleted and total RNA (*n*=14) (RNeasy Mini Kits, Qiagen, Valencia, CA) and DNA (*n*=8) (MasterPure complete DNA and RNA purification Kit, Epicentre, Madison, WI) was extracted and quality analyzed (Nanodrop, Bioanalyzer 2100, Agilent Technologies, Santa Clara, CA). For RNAseq, shotgun library construction (200 bp insert) was completed and then sequenced using a HiSeq2000 (91 bp pair-ended lane generating 2 Gb/sample, raw data) (Macrogen, Seoul, South Korea) and aligned to a human reference genome (UCSChg19). Transcriptomes were assembled, transcript abundance estimated (FPKM) and statistically significantly DEGs between paired specimens determined (2-fold up- or 0.5-fold downregulated expression) (Cufflinks and Cuffdiff) (Trapnell et al., 2010, 2012). The top 10 Hallmark gene sets were identified using MSigDB and IPA for the top 10 canonical pathways for DEGs ≥500 FPKM. Heat maps were generated using ClusVis [a web tool for visualizing clustering data (BETA)] (Metsalu and Vilo, 2015). To generate dendrograms, samples were clustered according to their gene expression profiles using the hclust function in R (R Development Core Team, 2013). Candidate fusion genes were identified with FusionCatcher Software (fusioncatcher.py 0.99.3e beta) (Nicorici et al., 2014). For Human Exome Capture sequencing, the Sure Select Target Enrichment System Capture Process was used with Illumina HiSeq2000 [91 bp paired end sequencing with SureSelect Human All Exon V4 (51 M) kit (50× on target coverage], followed by analysis of exome sequencing data using Burrows-Wheeler Aligner (Li and Durbin, 2010) to map data against UCSChg19 and SAMTOOLS (Li et al., 2009) for identification of SNPs and indels. RNAseq (http://www.ncbi.nlm.nih.gov/bioproject/412388) and DNA exome sequencing (http://www.ncbi.nlm.nih.gov/bioproject/407674) data are available in the Sequence Read Archive (SRA).

### RT-PCR, quantitative RT-PCR and FISH

RNA was extracted from cell pellets (RNeasy Mini Kit, Qiagen, Gaithersburg, MD), quantified (Nanodrop, ThermoFisher Scientific) and 1 μg total RNA was used to prepare cDNA (iScript™ cDNA Synthesis Kit, Bio-Rad, Hercules, CA). RT-PCR was performed using specific primer sets (Tables S2 and S3) and GoTaq^®^ Green Master Mix (Promega, Madison, WI) (30-40 cycles: 1 min: 95°C, 45 S-1 min: 55-60°C, 2 min: 72°C). Products were visualized using ethidium bromide staining after electrophoresis on agarose gels (Bio-Rad Universal Hood II, Bio-Rad). Candidate PCR products were extracted (QIAquick^®^ Gel Extract Kit, Qiagen) and sequenced (GENEWIZ, South Plainfield, NJ). For quantitative RT-PCR (qRT-PCR), 1 μg total RNA was converted to cDNA using Taqman reverse transcription reagents (Applied Biosystems, ThermoFisher Scientific) and 2.5 µl of the resulting cDNA used for each 20 μl reaction volume containing 10 μl 2× TaqMan^®^ Fast Universal PCR Master Mix (Applied Biosystems), 6.5 μl nuclease-free water, and 1 μl Taqman primers for *AREG* (4331182, ThermoFisher Scientific) or control 18 s (ThermoFisher Scientific). A Biosystems StepOne™/StepOnePlus™ Real-Time PCR System (Applied Biosystems) was used with 2 min: 50°C, 10 min: 95°C, and 40 cycles (15 s: 95°C, 1 min: 60°C). Data were analyzed by StepOne™ Software 2.3 (Applied Biosystems). For FISH, GUMC220/221 primary cells cultured in F medium+Y-27632 were detached from flasks by trypsinization with 0.05% trypsin and treated with hypotonic solution (0.075 M KCl), fixed on slides (3:1 methyl alcohol and glacial acetic acid), pretreated [Nonidet P-40, 20× saline-sodium citrate (SSC), and distilled water] at 37°C for 30 min and then dehydrated in an ascending series of ethanol solutions (70%, 80%, 95%) for 2 min each. FFPE sections were baked on slides overnight at 60°C to adhere tissue, and deparaffinized by consecutive 10-min xylene washes before being rehydrated through 100% ethanol incubation for 10 min at room temperature (RT). Tissues on the slides were permeabilized and digested using the Abbott tissue digestion kit containing pepsin (Naperville, IL), according to manufacturer's instructions, and washed. Probes for *KRT14*-20-RE (Empire Genomics, Buffalo, NY) covering 180 Kb of chromosome 17q21.2 including the entire *KRT14* gene, and *KRT5*-20-GR (Empire Genomics, Buffalo, NY) covering 151 Kb of chromosome12q13.13 including the entire *KRT5* gene, were denatured at 74°C for 10 min, mixed with hybridization buffer and immediately transferred to an ice bucket. Denatured probes were added to sections, and then slides were sealed with rubber cement and co-denatured for 8 min on a HYBrite heat plate (Vysis, Naperville, IL) at 85°C for 8 min, before incubation for 16-24 h at 37°C. Coverslips were then removed and slides were washed in 2× SSC hybridization buffer for 2 min at 73°C and transferred to 2× SSC at RT for 5 min. Slides were air dried in the dark for 1 h in an upright position, counterstained with 4′,6-diamidino-2-phenylindole (DAPI) (Vector Laboratories, Inc., Burlingame, CA) and viewed on an Axioscope fluorescence microscope (Zeiss, Oberkochen, Germany) and imaged with Applied Imaging Cytovision software (Pittsburgh, PA).

### Histology, IHC and western blotting

For hematoxylin and eosin (H&E) and IHC examination, FFPE 5-μm sections of excised original tissue (T) with identifiers removed, xenografts (X), Matrigel cultures (M) and primary cell pellets (P), prepared after fixation in 10% buffered formalin (Fisher Scientific, Hampton, NH) overnight at 4°C, were obtained from the HTSR. For IHC, antigen retrieval was performed using 10 mM citrate pH 6.0 buffer, EDTA pH 8.0 (home made), Tris/EDTA pH 9.0 (Genemed, South San Francisco, CA), and/or Envision FLEX Target Retrieval Solution Low pH (Agilent Technologies). For western blotting (WB), cell lysates were collected using radioimmunoprecipitation assay buffer (RIPA) (ThermoFisher Scientific) supplemented with Halt Protease and Phosphatase Inhibitor Cocktail (1:100) (ThermoFisher Scientific), sonicated, clarified by centrifugation (10 min), concentration measured (Pierce^®^ 660 nm Protein Assay Reagent, ThermoFisher Scientific) and 8 µg protein loaded with 2× Laemmli Sample Buffer (Bio-Rad) with 0.05 2-Mercaptoethanol (BME) on 10% gels (Bio-Rad) followed by transfer to PVDF membrane (Bio-Rad). Membranes were blocked with 7% bovine serum albumin (BSA) (Santa Cruz Biotechnology) prepared in 1× Tris-buffered saline, Tween 20 (TBST) [100 ml 10× TBS (Bio-Rad)+1 ml 100% Tween 20 (Fisher)+890 distilled water] for 1 h at RT, incubated overnight at 4°C with primary antibodies in 5% BSA in 1× TBST, washed three times with 1× TBST, incubated with appropriate polyclonal horseradish peroxidase (HRP)-conjugated secondary antibodies (1:5000 dilution in 1× TBST) for 1 h at RT, and then visualized by chemiluminescence (HyGLO Chemiluminescent HRP Antibody Detection Reagent, Denville Scientific, South Plainfield, NJ). Representative full-length WB images for each antibody used are shown (Fig. S5). Representative IHC images for each primary antibody on positive control tissue with (positive control) and without (reagent control) primary antibody are shown (Fig. S6). Positive control tissue was included with and without primary antibody in every experimental set. Optimal dilutions were established for each tissue type. Primary antibodies, cultures/assays and dilutions, antibody validation profiles and positive control tissues were as follows: AREG (HPA008720, Sigma-Aldrich); T (1:150), X (1:150), P (1:600), M (1:75); HPA (https://www.proteinatlas.org/); breast cancer (BrCa). EGFR (ab52894, Abcam, Cambridge, MA); T (1:75), P (1:200), X (1:200), M (1:100); CiteAb (https://www.citeab.com/), colon cancer (CoCa). p-EGFR [2236, Cell Signaling Technology (CST)]; T (1:100), P (1:1000), X (1:150), M (1:100); CiteAb; CoCa. Pan-AKT (4691, CST); WB (1:1000); Antibodypedia (https://www.antibodypedia.com/). p-AKT (Ser473) (4060, CST); T, P, X and M (1:30), WB (1:2000); Antibodypedia; CoCa. p-Akt (The308) (13038, CST); WB (1:1000); CiteAb. mTOR (2972, CST); WB (1:1000); CiteAb. p-mTOR (2976, CST); T, P, X and M (1:150); CiteAb; CoCa. p-mTOR (5536, CST); WB (1:1000); CiteAb; CoCa. GSK3α/β (5676, CST); WB (1:1000); Antibodypedia. p-GSK3α/β (9331, CST); WB (1:1000); Antibodypedia. β-Actin (3700, CST); WB (1:1000); Antibodypedia. Cytokeratin (KRT) 5 (PRB-160P, Covance, Gaithersburg, MD); T (1:150), P (1:2000), M (1:200); Antibodypedia; BrCa. KRT14 (PRB-155P, Covance); T (1:150), P (1:2000), M (1:100); Antibodypedia; BrCa. KRT8 (ab59400, Abcam); T and P (1:75), M (1:100); CiteAb; BrCa. KRT18 (4548, CST); T and P (1:200); M (1:200); Antibodypedia; BrCa. p53 (OP33) (PAb1620, Calbiochem-EMD Millipore, Billerica, MA); T, P and M (1:40); CiteAb; skin melanoma. Human mitochondrial protein (MAB1273, clone 113-1, EMD Millipore); X (1:100); CiteAb; BrCa. Secondary antibodies used were as follows: for WB, HRP-conjugated SC-2005 (goat anti-mouse, 1:5000), SC-2004 (goat anti-rabbit, 1:5000), SC-2020 (donkey anti-goat, 1:5000) (Santa Cruz Biotechnology); for IHC, avidin-biotin complex BA-1000 (goat anti-rabbit), BA-9200 (mouse) (Vector Laboratories, Burlingame, CA), HRP-conjugated K4001 (mouse), K4003 (rabbit) (DAKO Envision+, Agilent Technologies). A board-certified pathologist (B.V.K.) read histology and IHC blinded to antibody identity. Histology images were taken using a Nikon Eclipse E800 Microscope/ NIS-Elements BR 4.30.02 64-bit software (Nikon Instruments Inc., Melville, NY). Western blot images were taken by an Amersham™ Imager 600 (GE Health Care Life Sciences, Pittsburgh, PA) and quantified using ImageJ (http://imagej.nih.gov.proxy.library.georgetown.edu/ij). Relative expression levels were determined after normalizing total protein to actin signals obtained from the same membrane and phosphorylated protein to corresponding total protein. Means and standard errors of the mean were calculated, and data statistically analyzed and plotted using Prism 6.0 (GraphPad Software, La Jolla, CA). Each western blot was performed three times.

### Chemosensitivity and cell survival analyses

GUMC220/221 and MDA-MB-453 cells were plated in 96-well plates (5×10^4^ cell /100 µl of CM+Y, CM, or EpiC) for either 2D (VWR, Radnor, PA) or 3D (round-bottom low-attachment plates) culture. For 2D culture, cells were incubated overnight at 37°C with 5% CO_2_, followed by removal of the initial plating medium and replacement with medium containing different concentrations of MK2206 2HCI (MK2206) (1.2-20 μM), GSK690693 (2.5-40 μM), doxorubicin (0.3-5 μM) or vehicle-only dimethyl sulfoxide (DMSO) control. For 3D culture, cells were incubated overnight at 37°C with 5% CO_2_ followed by addition of MK2206 (1.2-40 μM), GSK690693 (1.2-40 μM), doxorubicin (0.3-10 μM) or vehicle-only (DMSO) control to the medium, with final concentration calculated for total well volume. Stock solutions (10 mM) of MK2206, GSK690693 and doxorubicin (Selleckchem, Houston, TX), was prepared in DMSO (VWR). Cell viability was measured using CellTiter-Glo^®^ Luminescent Cell Viability Assay (Promega, Madison, WI) according to the manufacturer's instructions after 3 (2D cultures) or 5 (3D cultures) days using Veritas microplate luminometer turner biosystems and GloMax^®^-96 Microplate Luminometer Software (Promega). Each experiment included three cell/condition technical replicates and each experiment was completed three times. DMSO luminescence values were normalized to nontreated wells and drug-treated wells normalized to corresponding DMSO wells.

### Statistics

Two-tailed Fisher’s exact test was used to compare the frequency distribution of the top 10 Hallmark pathways. Two-tailed, unpaired Student's *t*-test was used to analyze AREG qRT-PCR results. One-tailed, one-way ANOVA was to compare drug treatment response in primary cells. Welch-corrected, one-tailed, unpaired Student's *t*-test was used for western blot analyses. Data (mean±s.e.m.) were calculated and plotted using GraphPad Prism 6.0 (La Jolla, CA).

## Supplementary Material

Supplementary information
