# Supplementary material for: Expanding primary cells from mucoepidermoid and other salivary gland neoplasms for genetic and chemosensitivity testing
Source: Dis Model Mech. 2018 Jan 1;11(1):dmm031716. doi: 10.1242/dmm.031716 (PMC5818080; doi:10.1242/dmm.031716)
Supplement: Supplementary information [file dmm-11-031716-s1.pdf]

Table S1: Primary cell cultures established from salivary gland tissue.

Pathology	Salivary Gland	Race	Age	Sex	Primary Culture Identifiers
**Mucoepidermoid carcinoma**	Sublingual gland	White/Caucasian	56	F	GUMC220 GUMC221
**Carcinoma ex pleomorphic adenoma**	Parotid gland	Unknown	55	M	GUMC374 GUMC378
**Squamous cell carcinoma**	Sublingual gland	White/Caucasian	65	F	GUMC366 GUMC360
**Metastatic poorly differentiated squamous cell carcinoma**	Parotid gland	White/Caucasian	70	M	GUMC264 GUMC265
**Metastatic squamous cell carcinoma**	Parotid gland	White/Caucasian	80	M	GUMC367
**Benign pleomorphic adenoma**	Parotid gland	White/Caucasian	36	F	GUMC572 GUMC573
**Benign pleomorphic adenoma**	Parotid gland	White/Caucasian	39	M	GUMC349 GUMC332
**Benign pleomorphic adenoma**	Parotid gland	Other	39	M	GUMC436 GUMC446
**Benign pleomorphic adenoma**	Parotid gland	Unknown	45	F	GUMC520
**Benign pleomorphic adenoma**	Submandibular gland	White/Caucasian	68	M	GUMC299 GUMC284
**Diffuse large B cell lymphoma**	Submandibular gland	Black/African American	74	F	GUMC311 GUMC312
**Benign ductal squamous metaplasia**	Submandibular gland	Unknown	31	M	GUMC224

M: Male. F: Female. GUMC: Georgetown University Medical Center.

Disease Models & Mechanisms • Supplementary information

Table S2: Secondary *in vitro* culture and xenograft outcomes.

Primary Culture Identifier	Culture Condition / Pathology	2D				3D				
		CM+Y	CM	EpiC	Mammo-Cult	Soft agar F+Y	Matrigel F+Y	Low-adherent plate (CM+Y, CM, EpiC)	Xenograft Development: Mammary	Xenograft Development: Flank
**GUMC220**	Mucoepidermoid Carcinoma	Yes	Yes	Yes	Yes	No	Yes	Yes	1/8 injected sites	2/4 injected sites
**GUMC221**									2/8 injected sites	0/4 injected sites
**GUMC264** **GUMC265**	Metastatic poorly differentiated squamous cell carcinoma	Yes	Yes	Yes	ND	No	ND	ND	0/4 injected sites	0/2 injected sites
**GUMC367**	Metastatic squamous cell carcinoma	Yes	Yes	Yes	ND	No	ND	ND	0/4 injected sites	0/2 injected sites

Growth in specified condition is indicated by yes. No growth in specified condition is indicated by no. ND: Not done. CM+Y, conditioned media + ROCK inhibitor (Y-27632); CM, conditioned media without ROCK inhibitor (Y-27632); EpiC, EpiCult™-C Human Medium; Mammocult, Mammocult™ Human Medium Kit. F+Y, F-12 nutrient mix + ROCK inhibitor (Y-27632).

Disease Models & Mechanisms • Supplementary information

Table S3. Number of statistically significantly differentially expressed genes (DEGs) between paired primary culture samples from two different geographical regions of tumors.

Primary Culture Identifier	Number of DEGs Significantly Upregulated[1]	Total Number Significant DEGs	Total number of genes >5 FPKM	Pathology
**GUMC220**	48	66	26006	Mucoepidermoid carcinoma
**GUMC221**	18			
**GUMC264**	0	0	26006	Metastatic poorly differentiated squamous cell carcinoma
**GUMC265**	0			
**GUMC367**	0	0	26006	Metastatic squamous cell carcinoma
**GUMC374**	0	0	26006	Carcinoma ex pleomorphic adenoma
**GUMC378**	0			
**GUMC572**	19	84	26006	Benign pleomorphic adenoma
**GUMC573**	65			
**GUMC332**	0	18	26006	Benign pleomorphic adenoma
**GUMC349**	18			
**GUMC436**	57	98	26006	Benign pleomorphic adenoma
**GUMC446**	41			
**GUMC284**	28	68	26006	Benign pleomorphic adenoma
**GUMC299**	40			
**GUMC311**	12	14	26006	Diffuse large B cell lymphoma
**GUMC312**	2			

Disease Models & Mechanisms • Supplementary information

Table S4. SNPs with published association with increased cancer risk.

**Salivary Neoplasm**	**Muco-epidermoid Carcinoma**		**Malignant Pleomorphic Adenoma**		**Benign Pleomorphic Adenoma**		**Benign Pleomorphic Adenoma**		**Reference(s)**	**Cancer Type(s) Reported**
**Cell Culture Identifier**	220	221	374	378	436	446	572	573		
***BRCA2* rs144848**	Het	Het					Het	Het	(Jiao et al. 2012) (Guo et al. 2014) (Johnson et al. 2007)	Non-Hodgkin's Lymphoma; Breast
***TP53* rs1042522**	Het	Het							(Tian et al. 2017) (Johnson et al. 2007) (Cheng et al. 2014)	Colorectal, Breast, Gastric
***AURKA* rs2273535**	Het	Het			Hom	Hom			(Ewart-Toland et al. 2005) (Chou et al. 2017) (Dai et al. 2014)	Colorectal, Breast, Oral
***RET* rs1800858**	Het	Het	Hom	Hom			Hom	Hom	(Huang and Yang 2015)	Thyroid
***NQO1* rs1800566**			Het	Het					(Dong et al. 2016) (Hu et al. 2014)	Hepatocellular, Gastric
***XRCC3* rs861539**					Het	Het			(Cui et al. 2016) (Yan et al. 2016)	Naso-pharyngea, Thyroid
***RNASEL* rs486907**					Hom	Hom			(Alvarez-Cubero et al. 2016) (Agalliu et al. 2010)	Prostate
***EPHX1* rs2234922**					Het	Het	Het	Het	(Xu et al. 2015)	Lung
ADH1B rs1229984	Het	Het	Hom	Hom					(Tanaka et al. 2010)	Esophageal

SNP: single-nucleotide polymorphism. Het: Heterozygous. Hom: Homozygous.

Table S5: RT-PCR for primer sets for known MEC-associated fusion genes.

Gene name	Primers	Product size	Reference
CRTC1-MAML2_1	F_5-GCCTTCGAGGAGGTCATGA-3<br>R_5-CTTGCTGTTGGCAGGAGA-3	105bp	(Fehr et al. 2008)
CRTC1-MAML2_2	F_5-TTCGAGGAGGTCATGAAGGA-3<br>R_5-TTGCTGTTGGCAGGAGATAG-3	101bp	(Chen et al. 2014)
CRTC3-MAML2	F_5-CGCGGAAGTTCAGTGAGA-3<br>R_5-CTTGCTGTTGGCAGGAGA-3	155bp	(Fehr et al. 2008)
WT-MAML2	F_5-GTAGCAATAATGGTGGCAGT-3<br>R_5-CTTGCTGTTGGCAGGAGA-3	155bp	(Fehr et al. 2008)
EWS-POU5F1	F_5-AGCCTGTCCAGGGGTATGGCACT-3<br>R_5-TTCCTAGAAGGGCAGGCACCTC-3	800bp	(Möller et al. 2008)
GAPDH	F_5-CAATGACCCCTTCATTGACC-3<br>R_5-GACAAGCTTCCCGTTCTCAG-3	107bp	(Chen et al. 2014)

F: Forward. R: Reverse.

Table S6. Candidate fusion sequences identified using FusionCatcher (Nicorici et al., 2014 preprint)**.**

Gene_1_symbol(5end_fusion_partner)	Gene_2_symbol(3end_fusion_partner)	Fusion finding method	Fusion sequence
KRT14	***KRT5***	BOWTIE+ BLAT	TGGGGGAGGATATGGTGGTGGCCTTGGTGCTGGCTTGGGTGGTGGCTTTG****GTGGTGGCTTTGGGCTCGGTGGCGGAGCTGGCTTTGGAGGTGGCTTCGGT**
KRT14	***KRT5***	BOWTIE+ STAR	TTTGGGGGAGGATATGGTGGTGGCCTTGGTGCTGGCTTGGGTGGTGGCTT****TGGTGGTGGCTTTGGGCTCGGTGGCGGAGCTGGCTTTGGAGGTGGCTTCG**
KRT14	***KRT5***	BOWTIE+ STAR	AGCAGCTTTGGTAGTGGCTTTGGGGGAGGATATGGTGGTGGCCTTGGTGC****TGGCGGTGGTGGTGGCTTCGGCAGGGTCAGCCTTGCGGGTGCTTGTGGAG**
KRT14	***KRT5***	BOWTIE+ BLAT	TGGGGGAGGATATGGTGGTGGCCTTGGTGCTGGCTTGGGTGGTGGCTTTG****GAGGTGGCTTCGGTGGCCCTGGCTTTCCTGTCTGCCCTCCTGGAGGTATC**
KRT5	***KRT14***	BOWTIE+ STAR	CTGGAGGCGGCTATGGCTTTGGAGGTGGTGCCGGTAGTGGATTTGGTTTC****GGTGGCTTTGCTGGTGGTGATGGGCTTCTGGTGGGCAGTGAGAAGGTGAC**
KRT5	***KRT14***	BOWTIE+ BLAT	TGGTGGTGGCTTCGGCAGGGTCAGCCTTGCGGGTGCTTGTGGAGTGGGTG****GCTATGGCGGTGGCTTCAGCAGCAGCAGCAGCAGCTTTGGTAGTGGCTTT**
KRT5	***KRT14***	BOWTIE+ STAR	GTGGTGGTGGCTTCGGCAGGGTCAGCCTTGCGGGTGCTTGTGGAGTGGGT****GGCTATGGCGGTGGCTTCAGCAGCAGCAGCAGCAGCTTTGGTAGTGGCTT**
KRT14	***NCL***	BOWTIE+ BLAT	TGGAAGCCGACATCAATGGCCTGCGCAGGGTGCTGGACGAACTGACCCTG****GCCAAAGCTGCCAAGGAGGCCATGGAAGACGGTGAAATTGATGGAAATAA**
KRT14	***NCL***	BOWTIE+ STAR	TGGAAGCCGACATCAATGGCCTGCGCAGGGTGCTGGACGAACTGACCCTG****GCCAAAGCTGCCAAGGAGGCCATGGAAGACGGTGAAATTGATGGAAATAA**

Disease Models & Mechanisms • Supplementary information

Table S7. *KRT14-KRT5* primer sets.

Primer pair names	Primer sequences	Product size
KRT5-KRT13	F_5-GTGCCGGTAGTGGATTTGGT-3<br>R_5-CCAGCAAAACCCCCACCAAA-3	100bp
KRT5-KRT14	F_5-TGGATTTGGTTTCGGCGGTG-3<br>R_5-GTCATTGAGGTTCTGCATGG-3	100bp
*KRT5-KRT14*_1	F_5-CTGGAGGCGGCTATGGCTTT-3<br>R_5-GTCACCTTCTCACTGCCCAC-3	100bp
*KRT5-KRT14*_2	F_5-GTGGTGGTGGCTTCGGCAGG-3<br>R_5-AAGCCACTACCAAAGCTGCT-3	100bp
KRT14-NCL	F_5-TGGAAGCCGACATCAATGGC-3<br>R_5-TTATTTCCATCAATTTCACC-3	100bp
KRT14-KRT5-1	F_5-TGGGGGAGGATATGGTGGTG-3<br>R_5-ACCGAAGCCACCTCCAAAGC-3	100bp
KRT14-KRT5-2	F_5-AGCAGCTTTGGTAGTGGCTT-3<br>R_5-CTCCACAAGCACCCGCAAGG-3	100bp
KRT14-KRT5-3	F_5-TGGGGGAGGATATGGTGGTG-3<br>R_5-GATACCTCCAGGAGGGCAGA-3	100bp
KRT14-KRT5-4	F_5-CCACCTGCAGCCGCCAGTTC-3<br>R_5- CCGAAGCCACCTCCAAAGCC-3	300bp

Disease Models & Mechanisms • Supplementary information

Supplementary Figure 1

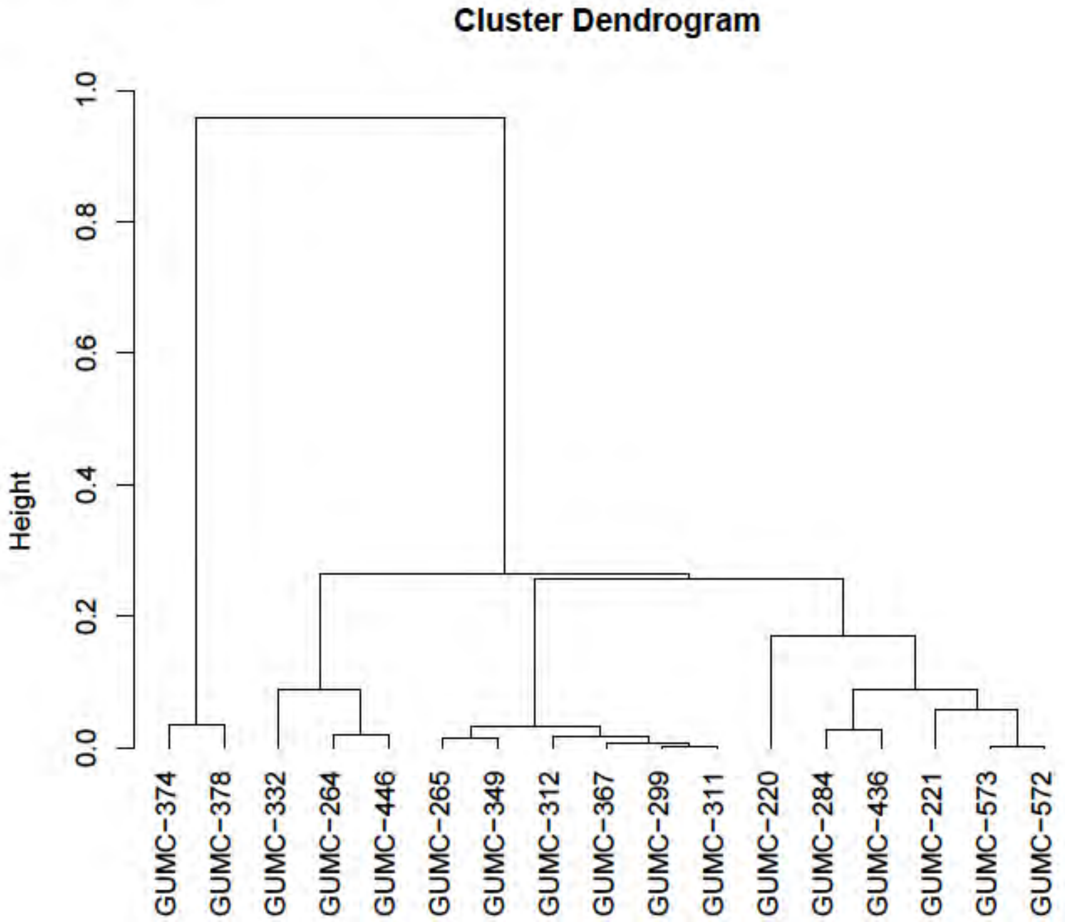

Dendrogram illustrating hierarchical clustering of paired culture transcriptomes. Samples were clustered according to their gene expression profiles using the hclust function in R (http://www.R-project.org). GUMC-374/378: Carcinoma ex pleomorphic adenoma. GUMC-332/349: Benign pleomorphic adenoma. GUMC-264/265: Metastatic poorly differentiated squamous cell carcinoma. GUMC-446/436: Benign pleomorphic adenoma. GUMC-312/311: Diffuse Large B Cell Lymphoma. GUMC-367: Metastatic squamous cell carcinoma. GUMC-299/284: Benign pleomorphic adenoma. GUMC-220/221: Mucoepidermoid carcinoma. GUMC-572/573: Benign pleomorphic adenoma.

Disease Models & Mechanisms • Supplementary information

Supplementary Figure 2

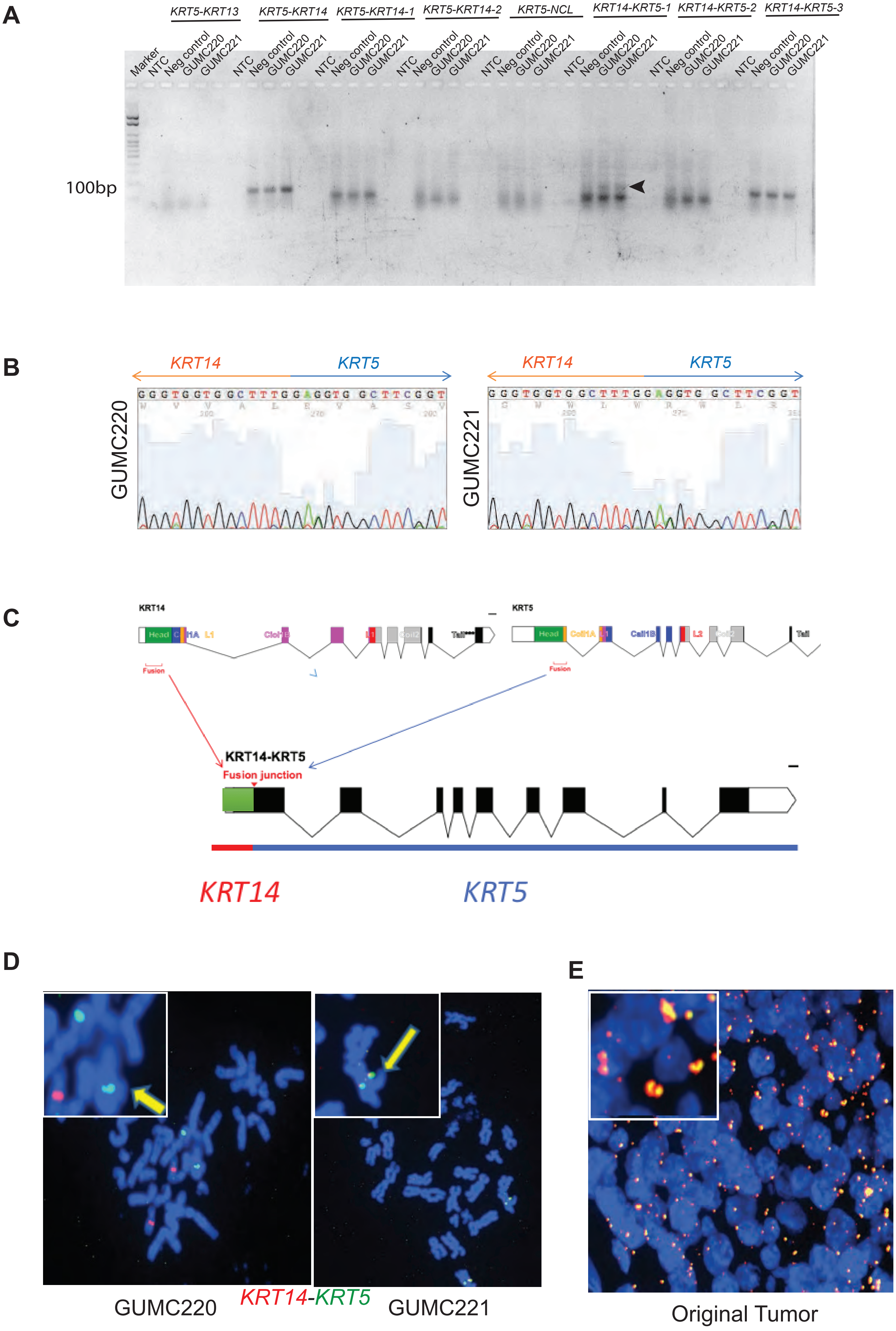

Disease Models & Mechanisms • Supplementary information

Supplementary Figure 2

***KRT14-KRT5* gene fusion analyses.** (A) Ethidium bromide stained agarose gel illustrating RT-PCR screening for eight primer sets, *KRT5-KRT13, KRT5-KRT14, KRT5-KRT14_1, KRT5-KRT14_2, KRT5-NCL, KRT14-KRT5_1, KRT14-KRT5_2, KRT14-KRT5_3,* targeting the potential fusion gene junction identified by FusionCatcher in GUMC220 and GUMC221. Arrowhead indicates PCR products isolated for sequencing from the *KRT14-KRT5_1* primer pair. (B) Base-calling sequencing electropherogram of the *KRT14-KRT5_1* RT-PCR amplicon illustrating potential *KRT14-KRT5* fusion gene junction sequence in GUMC220 and GUMC221. (C) Predicted exon 1 site of *KRT14-KRT5* fusion gene junction within predicted exon structure of fusion gene shown with *KRT14* gene exon structure (left) and *KRT5* gene exon structure (right). (D) Representative fluorescence images illustrating *KRT14* (red) and *KRT5* (green) FISH performed on chromosomes harvested from CRC-cultured GUMC220 and GUMC221 cells. FISH performed independently three times on each culture. Images taken at 100X. Top left insert shows magnified images. Yellow arrows indicate representative examples of green (KRT5) signal on paired chromosomes as positive control. Co-localization of red and green probes could not be confirmed in the spreads of the cell cultures examined. (E) Representative fluorescence images illustrating *KRT14* (red) and *KRT5* (green) FISH performed on formalin fixed paraffin embedded (FFPE) section from the mucoepidermoid carcinoma GUMC220/221 were derived from demonstrating areas of red and green probe overlay in tissue sections (yellow color). FISH performed independently three times. Top left insert shows magnified image. Images taken at 100X.

Disease Models & Mechanisms • Supplementary information

Supplementary Figure 3

A

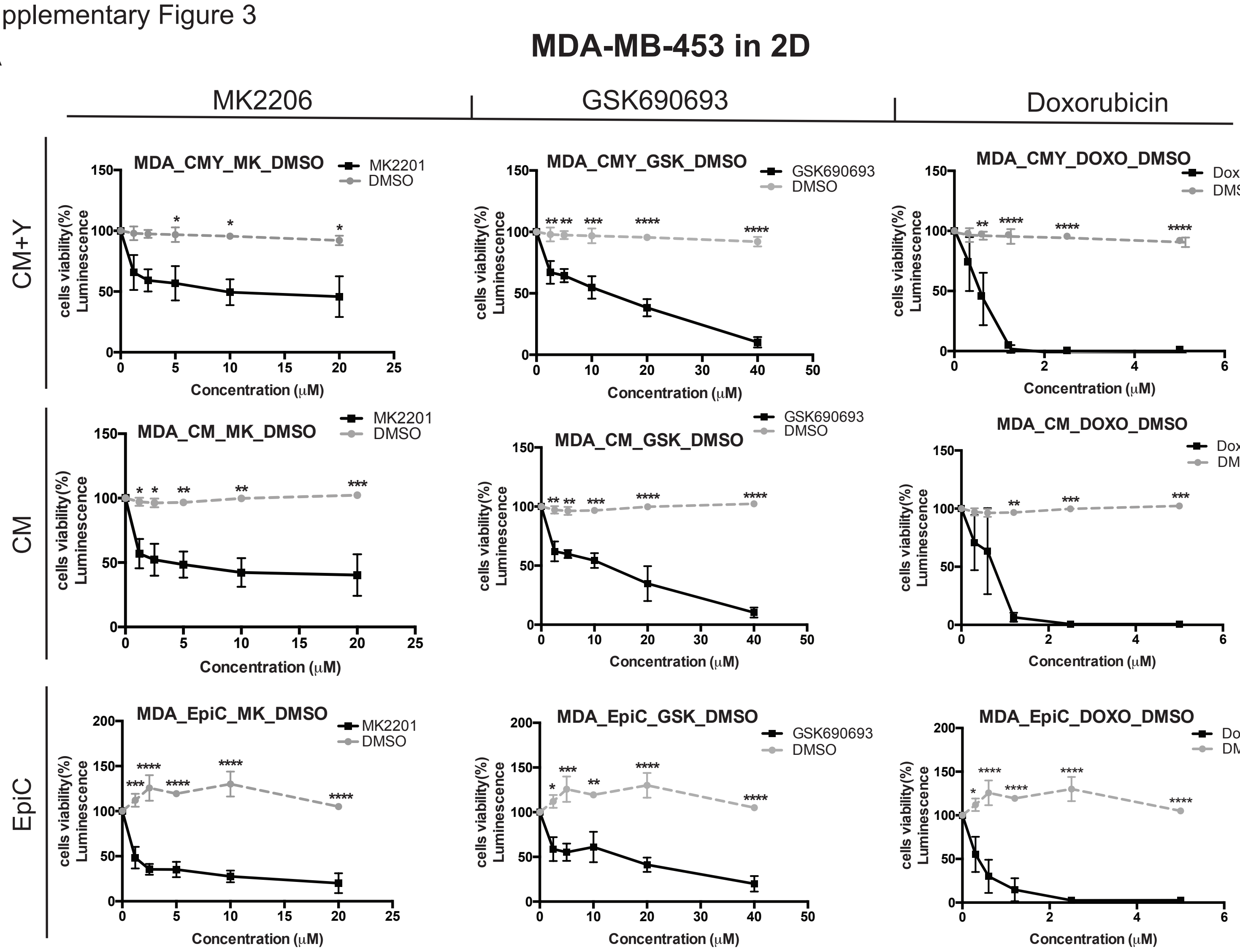

B

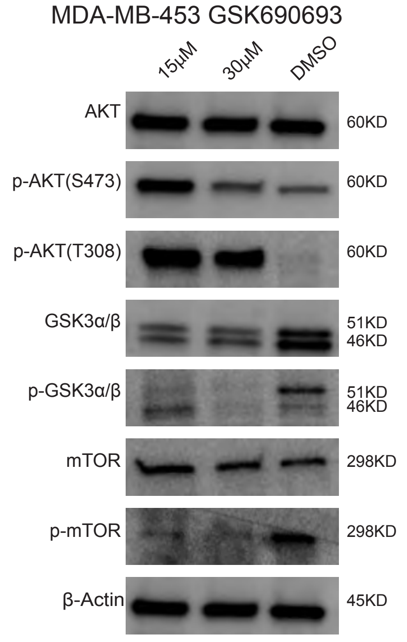

Viability of MD-MB-453 2D cell cultures exposed to MK2206, GSK690639 and doxorubicin in CM+Y, CM and EpiC media with western blot analyses of steady state levels of AKT, p-AKT, GSK3α/β, p- GSK3α/β, mTOR, and p-mTOR following exposure to GSK690693 in CM+Y.

(A) Cell viability measured after three days for MD-MB-453 cells under three different 2D culture media (CM+Y, CM, and EpiC) at a range of concentrations in the presence of left, MK2206 (1.2μM-20μM, black line) compared to DMSO (vehicle control, gray line), middle, GSK690639 (2.5μM- 40μM, black line) compared to DMSO (vehicle control, gray line), right, doxorubicin (0.3μM-5μM, black line) compared to DMSO (vehicle control, gray line). Mean±s.e.m. shown. One DMSO vehicle control for each medium used each experiment performed (dotted grey line), n=3 replicates/medium/drug/concentration. * $p \leq 0.05$, ** $p \leq 0.01$, *** $p \leq 0.001$, **** $p \leq 0.0001$, One-way ANOVA, one-tailed. (B) Representative western blots of steady state levels of AKT, p-AKT(S473), p-AKT(T308), GSK3α/β, p-GSK3α/β, mTOR, p-mTOR, and β-Actin from MDA-MB-453 cells under 2D CM+Y culture exposed to two different concentrations of GSK690693 (15μM and 30μM) compared with DMSO-only control. Protein lysates collected after 3 days of drug exposure. MW in kiloDaltons (kDa) for each protein indicated.

Disease Models & Mechanisms • Supplementary information

Supplementary Figure 4

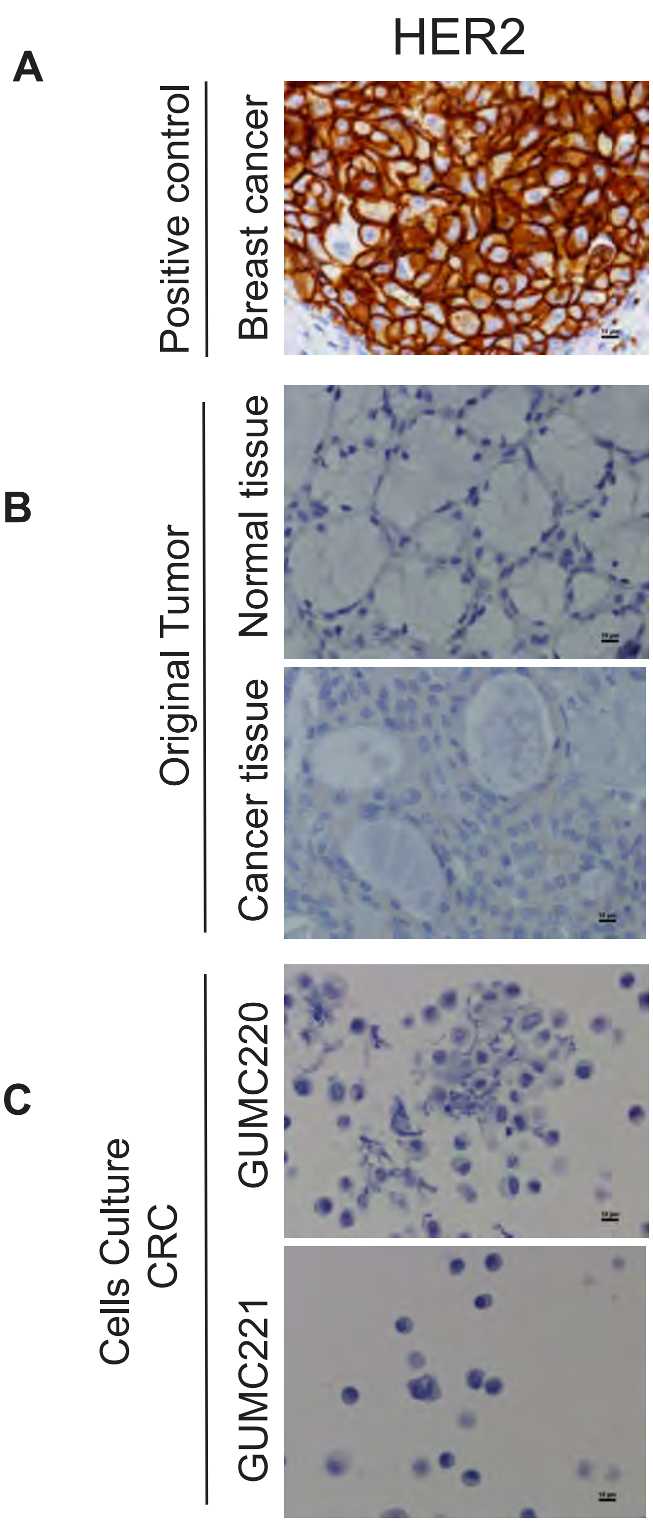

Immunohistochemistry for HER2.

(A) Representative HER2 IHC image of human breast cancer tissue used as a positive control. (B) Representative HER2 IHC images of adjacent normal tissue (top) and mucoepidermoid carcinoma cancer tissue (bottom). (C) Representative HER2 IHC images of GUMC220 (top) and GUMC221 (bottom) cell pellets from CRC cultures.

Disease Models & Mechanisms • Supplementary information

Supplementary Figure 5

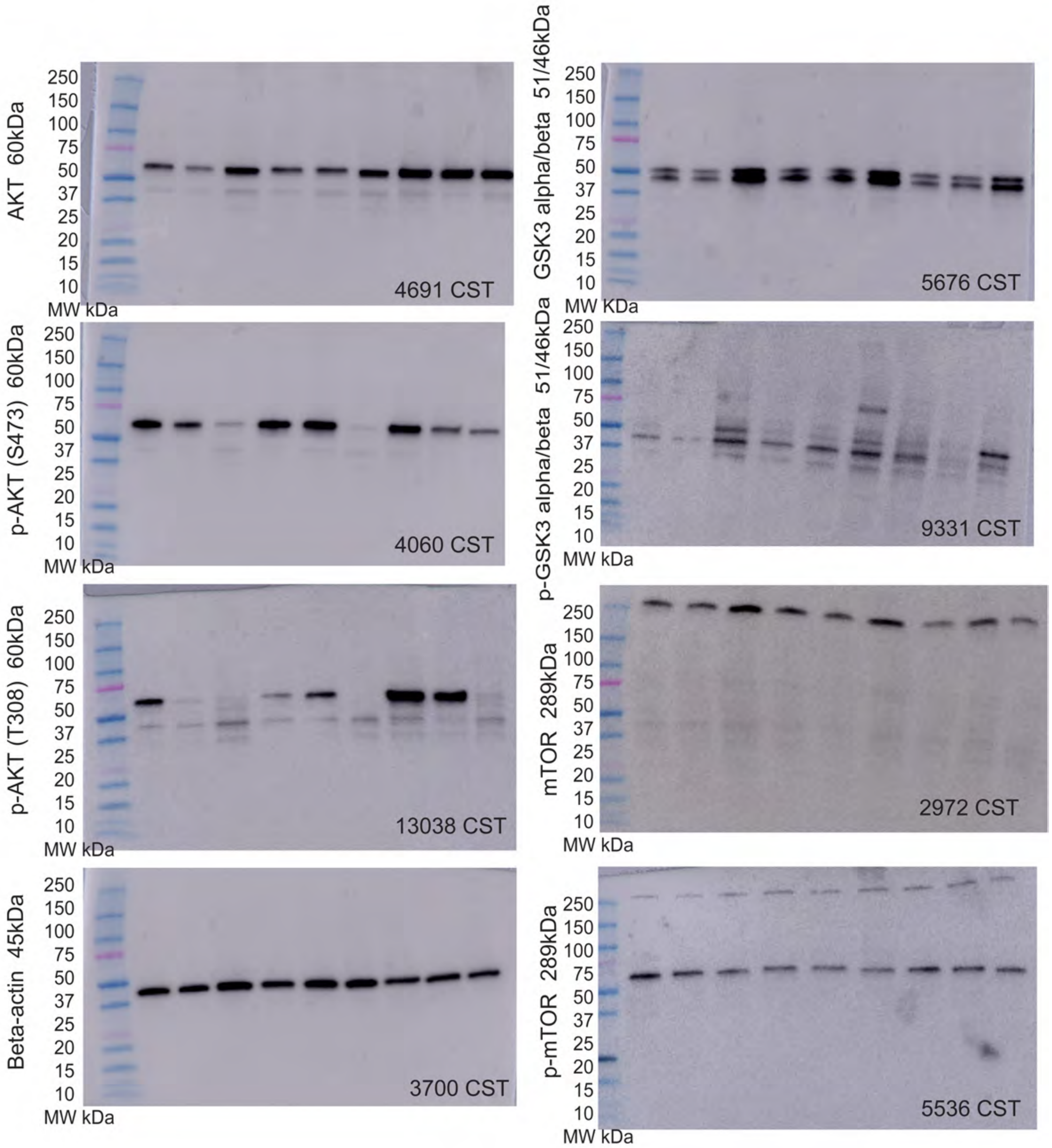

Supplementary Figure 5. Full-length images of western blots. Representative full-length image shown for each antibody used for western blotting. Antibody catalog number and supplier at bottom right of each image. Protein targeted by each antibody and the molecular weight in kiloDaltons (kDa) of that protein indicated to left of each image. Molecular weights (MW) (kDa) of size markers used on each blot indicated. CST: Cell Signaling Technology.

Disease Models & Mechanisms • Supplementary information

Supplementary Figure 6

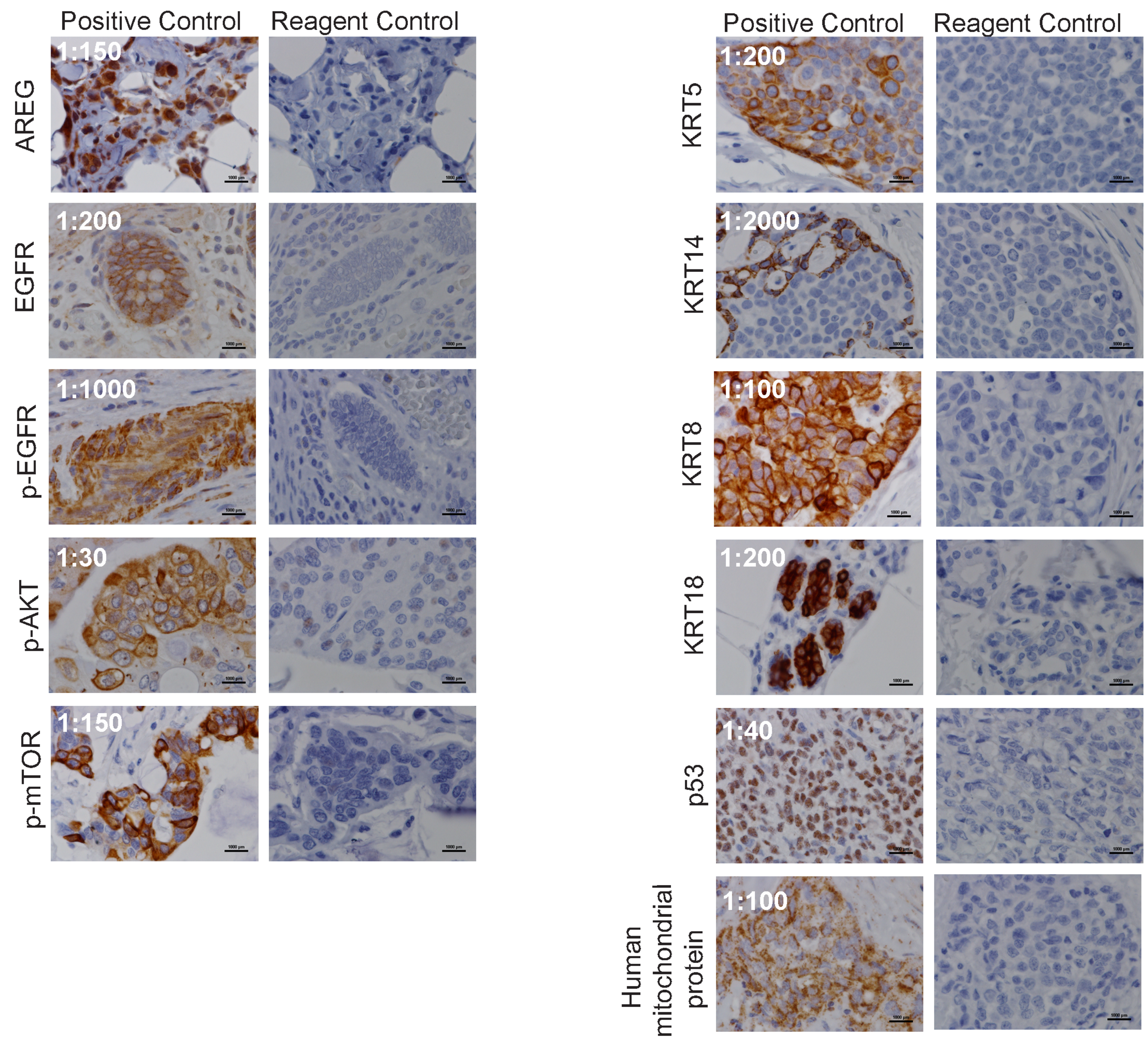

Positive control and reagent control immunohistochemistry.

Representative images of tissue used as positive controls for immunohistochemistry with (positive control) and without (reagent control) primary antibody. Protein targeted by each antibody indicated to left of each image. Primary antibody dilution shown indicated top left corner of each image. Positive control tissues and antibodies used specified in Materials and Methods. Images taken at 60X.

Disease Models & Mechanisms • Supplementary information
